# Quantitative phase imaging techniques for measuring scattering properties of cells and tissues: a review–part II

**DOI:** 10.1117/1.JBO.29.S2.S22714

**Published:** 2024-07-27

**Authors:** Neha Goswami, Mark A. Anastasio, Gabriel Popescu

**Affiliations:** a University of Illinois Urbana-Champaign, Department of Bioengineering, Urbana, Illinois, United States; b University of Illinois Urbana-Champaign, Department of Electrical and Computer Engineering, Urbana, Illinois, United States

**Keywords:** light scattering, digital holography, quantitative phase imaging

## Abstract

**Significance:**

Quantitative phase imaging (QPI) is a non-invasive, label-free technique that provides intrinsic information about the sample under study. Such information includes the structure, function, and dynamics of the sample. QPI overcomes the limitations of conventional fluorescence microscopy in terms of phototoxicity to the sample and photobleaching of the fluorophore. As such, the application of QPI in estimating the three-dimensional (3D) structure and dynamics is well-suited for a range of samples from intracellular organelles to highly scattering multicellular samples while allowing for longer observation windows.

**Aim:**

We aim to provide a comprehensive review of 3D QPI and related phase-based measurement techniques along with a discussion of methods for the estimation of sample dynamics.

**Approach:**

We present information collected from 106 publications that cover the theoretical description of 3D light scattering and the implementation of related measurement techniques for the study of the structure and dynamics of the sample. We conclude with a discussion of the applications of the reviewed techniques in the biomedical field.

**Results:**

QPI has been successfully applied to 3D sample imaging. The scattering-based contrast provides measurements of intrinsic quantities of the sample that are indicative of disease state, stage of growth, or overall dynamics.

**Conclusions:**

We reviewed state-of-the-art QPI techniques for 3D imaging and dynamics estimation of biological samples. Both theoretical and experimental aspects of various techniques were discussed. We also presented the applications of the discussed techniques as applied to biomedicine and biology research.

## 1 Introduction: Quantitative Phase Imaging

Quantitative phase imaging (QPI) has emerged as a highly sensitive imaging technique for non-invasive, label-free characterization of biological samples.^1^ QPI is an umbrella term that includes various varieties of interferometric-, holographic-, or intensity-based optical measurements that are capable of quantifying the interaction of light with matter without the addition of external reagents. Such imaging modalities produce an output image [two-dimensional (2D) (xy), three-dimensional 3D (xyz), or four-dimensional (4D) (xyzt)], in which the contrast is generated by the sample properties, such as refractive index (RI) variations, depth fluctuations, and anisotropy. This intrinsic contrast based on the scattering properties of a sample enables the long-term observation of samples in their inherent state without the addition of external reagents.^2^ Over the past decade, with increased interest from the biological community in 3D samples, such as organoids, spheroids, and embryos,^3^ new 3D imaging modalities have been actively developed.^4^^,^^5^ Optical imaging of such thick samples is challenging because of the sample’s highly scattering nature, which limits the depth of penetration of light and hence lowers the imaging contrast. Due to their larger size compared with single cells, these 3D samples need a larger number of axial scans, which increases the light dosage delivered to the sample, thereby increasing the risk of phototoxicity. As such, the need for label-free, QPI techniques is even more crucial in these applications because QPI requires lower illumination power, thereby reducing the effects of phototoxicity and eliminating photobleaching, and provides better depth sectioning.^6^

Non-invasive, label-free QPI techniques can enable the imaging of highly sensitive samples such as live-embryos. The introduction of machine learning to improve the reconstruction of 3D volumes can further enhance the capabilities of these imaging modalities by lowering toxicity. This can be achieved, for example, by providing fluorescence-like information without staining the sample or by relaxing data-acquisition requirements.^7^ The capability of estimating the 3D quantitative phase/RI tomograms through QPI techniques additionally may facilitate the identification of new biomarkers that can help in the biological characterization and/or disease diagnosis or progression in 3D.^7^

In part I of this review,^8^ we discussed the basic principles and theoretical formulations of QPI and 2D scattering. We also discuss topics that include 2D QPI techniques—spatial light interference microscopy (SLIM),^9^ diffraction phase microscopy (DPM),^10^ gradient light interference microscopy (GLIM),^11^ epi-illumination GLIM (Epi-GLIM),^12^ various realizations of digital holographic microscopy (DHM),^13^^,^^14^ and Hilbert phase microscopy (HPM).^15^ We reviewed the Fourier transform light scattering (FTLS)^16^ technique, which is a static light scattering method. Applications of these techniques were also surveyed.

In part II of this review, we address 3D QPI techniques and their applications and also discuss dynamic light scattering (DLS) techniques. We start with a theoretical description of 3D scattering followed by different tomographic techniques that include various configurations of optical diffraction tomography (ODT),^17^ variants of phase-resolved optical coherence tomography (PR-OCT),^18^^–^^20^ harmonic optical tomography (HOT),^21^ interferometric synthetic aperture microscopy (ISAM),^22^ computational adaptive optics for ISAM (CAO-ISAM),^23^ and tomographic phase microscopy (TPM).^24^ We review some of the principles of and developments in DLS measurements, such as DLS,^25^^–^^28^ diffusing-wave spectroscopy (DWS),^29^^,^^30^ and dispersion-relation phase spectroscopy (DPS).^31^ We conclude by presenting some applications of the imaging techniques presented in parts I and II related to the assessment of cellular and intracellular dynamics of biological samples.

## 2 3D Scattering

### 2.1 Optical Diffraction Tomography

In 1969, Wolf^32^^,^^33^ introduced the concept of diffraction tomography. Here, we describe the theoretical foundation for diffraction tomography, taking advantage of k-domain formulation.^33^

**Fig. 1:**
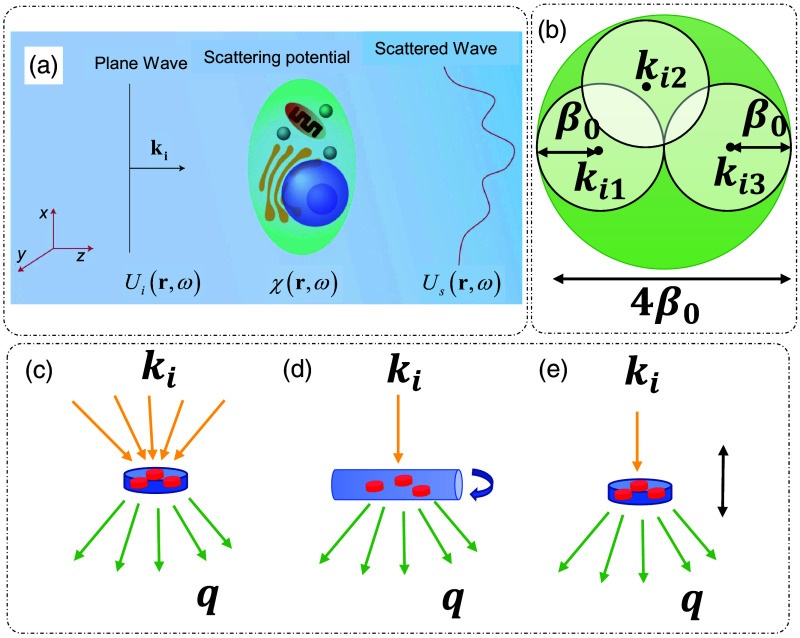
ODT. (a) Scattering geometry: a plane wave with incident wavevector $k_{i}$ illuminates the sample. χ(r,ω): scattering potential of the sample. $U_{i}(r,\omega )$: incident field. $U_{s}(r,\omega )$: scattered field. (b) Ewald’s limiting sphere, with $k_{i,n}$ indicating the n’th incident wavevector and $\beta_{0}$ being the free space wavevector. Main modes of ODT: (c) rotation of illumination beam, (d) rotation of sample, and (e) axial scanning of sample, q is the scattering wavevector. (a) Reproduced with permission from Ref. 34, © 2014, Springer Nature Limited.

Consider the inhomogeneous Helmholtz equation and the geometry shown in Fig. 1(a):

$$\nabla^{2}U(r,\omega )+\beta_{0}^{2}U(r,\omega )=-\beta_{0}^{2}[n^{2}(r,\omega )-1]U(r,\omega ),$$ (1)

where $\beta_{0}=\frac{2\pi }{\lambda }$ is the free space propagation constant, n(r,ω) is the RI distribution of the sample, and U is the total field comprising the incident and the scattered components. Let the scattering potential of the sample be denoted by $\chi (r,\omega )=\beta_{0}^{2}[n^{2}(r,\omega )-1]$, considering the surrounding media to be free space. Equation (1) is then expressed as^6^

$$\nabla^{2}U(r,\omega )+\beta_{0}^{2}U(r,\omega )=-\chi (r,\omega )U(r,\omega ).$$ (2)

Under the assumption of a weakly scattering sample, we invoke the first-order Born approximation, which implies that the field inside the sample is expressed as the incident field $U_{i}(r,\omega )$, and the scattered field is then expressed as^6^

$$\nabla^{2}U_{s}(r,\omega )+\beta_{0}^{2}U_{s}(r,\omega )=-\chi (r,\omega )U_{i}(r,\omega ).$$ (3)

Taking the 3D Fourier transform with respect to r, we obtain

$${\overset{˜}{U}}_{s}(k,\omega )=\frac{\overset{˜}{\chi }(k,\omega ){Ⓥ}_{k}{\overset{˜}{U}}_{i}(k,\omega )}{k^{2}-\beta_{0}^{2}}\text{,}$$ (4)

where ${Ⓥ}_{k}$ indicates a convolution in the spatial frequency (k) domain, which is the Fourier conjugate to the spatial (r) domain.

Assuming a plane wave incidence with the direction denoted by wavevector $k_{i}$, namely, $U_{i}(r,\omega )=e^{ik_{i}•r}$, Eq. (4) reduces to

$${\overset{˜}{U}}_{s}(k,\omega )=\frac{\overset{˜}{\chi }(k,\omega ){Ⓥ}_{k}\delta (k-k_{i},\omega )}{k^{2}-\beta_{0}^{2}}=\frac{\overset{˜}{\chi }(k-k_{i},\omega )}{k^{2}-\beta_{0}^{2}},$$ (5)

Equation (5) is decomposed into partial fractions as

$${\overset{˜}{U}}_{s}(k,\omega )=\frac{\overset{˜}{\chi }(k-k_{i},\omega )}{k_{z}^{2}-(\beta_{0}^{2}-k_{⊥}^{2})}=\frac{\overset{˜}{\chi }(k-k_{i},\omega )}{2\gamma (k_{⊥})}[\frac{1}{k_{z}-\gamma (k_{⊥})}-\frac{1}{k_{z}+\gamma (k_{⊥})}],$$ (6)

where $\gamma (k_{⊥})=\sqrt{\left(\beta_{0}^{2}-k_{⊥}^{2}\right)}$.

Taking inverse one-dimensional Fourier transform with respect to the z coordinate yields

$${\overset{˜}{U}}_{s}(k_{⊥},z,\omega )=\frac{iF_{k_{z}}^{-1}\left[\overset{˜}{\chi }(k-k_{i},\omega )\right]}{2\gamma (k_{⊥})}{Ⓥ}_{z}[e^{i\gamma (k_{⊥})z}\text{ sign}(z)+e^{-i\gamma (k_{⊥})z}\text{ sign}(-z)],$$ (7)

where $k_{⊥}=(k_{x},k_{y})$ is the transverse spatial frequency and $F_{k_{z}}^{-1}$ is the inverse Fourier transform operator in the $k_{z}$ domain. Selecting the forward traveling wave component and noting that $F_{k_{z}}^{-1}[\tilde{\chi }(k-k_{i},\omega )]=\tilde{\chi }(k_{⊥}-k_{i⊥},z,\omega )e^{ik_{iz}z}$, Eq. (7) simplifies to

$${\overset{˜}{U}}_{s}(k_{⊥},z,\omega )=i\frac{e^{i\gamma (k_{⊥})z}{Ⓥ}_{z}\overset{˜}{\chi }(k_{⊥}-k_{i⊥},z,\omega )e^{ik_{iz}z}}{2\gamma (k_{⊥})}\text{,}$$ (8)

where $k_{i⊥}=(k_{ix},k_{iy})$ denotes the transverse component of the incident field’s wavevector.

Convolution with a complex exponential yields a Fourier transform at a specified frequency. Hence, Eq. (8) reduces to

$${\overset{˜}{U}}_{s}(k_{⊥},z,\omega )=i\frac{e^{i\gamma (k_{⊥})z}\overset{˜}{\chi }(k_{⊥}-k_{i⊥},\gamma (k_{⊥})-k_{iz},\omega )}{2\gamma (k_{⊥})}.$$ (9)

The above equation is rearranged such that

$$\overset{˜}{\chi }(k_{⊥}-k_{i⊥},\gamma (k_{⊥})-k_{iz},\omega )=-2i\gamma (k_{⊥})e^{-i\gamma (k_{⊥})z}{\overset{˜}{U}}_{s}(k_{⊥},z,\omega ).$$ (10)

Equation (10) implies that the 3D spatial frequency information of the scattering potential can be recovered by measuring the 2D Fourier transforms of the scattered field at different axial locations, thus providing a solution for the inverse problem.^33^

Note that Eq. (10) contains no approximations other than the first-order Born. In other words, there are no angular approximations.

To recover Wolf’s^33^ description for scattering at large distances, i.e., in the far-zone, we express the scattered field in the spatial domain as

$$U_{s}(r,\omega )=\chi (r,\omega )e^{ik_{i}\cdot r}{Ⓥ}_{r}\frac{e^{i\beta_{0}r}}{r}\;=\int \chi (r^{\prime },\omega )e^{ik_{i}\cdot r^{\prime }}\frac{e^{i\beta_{0}|r-r^{\prime }|}}{\left|r-r^{\prime }\right|}d^{3}r^{\prime }.$$ (11)

Considering far-zone detection, such that $|r-r^{\prime }|\approx r-\frac{k_{s}}{\beta_{0}}\cdot r^{\prime }$ in the exponent and $\frac{1}{\left|r-r^{\prime }\right|}≃\frac{1}{r}$, where $k_{s}$ is the wavevector in the direction of the observation, Eq. (11) reduces to

$$U_{s}(r,\omega )=\frac{e^{i\beta_{0}r}}{r}\int \chi (r^{\prime },\omega )e^{i(k_{i}-k_{s})\cdot r^{\prime }}d^{3}r^{\prime }.$$ (12)

If we define momentum transfer^6^ or the scattering wavevector $q=k_{s}-k_{i}$, Eq. (12) becomes

$$U_{s}(r,\omega )=\frac{e^{i\beta_{0}r}}{r}\int \chi (r^{\prime },\omega )e^{-iq\cdot r^{\prime }}d^{3}r^{\prime }.$$ (13)

Thus, the scattered field is the 3D Fourier transform of the scattering potential evaluated at q, multiplied by the spherical wavelet $\frac{e^{i\beta_{0}r}}{r}$.

At a particular angle of incidence, the maximum extent of spatial frequencies covered by measuring the scattered field in different angular directions (governed by $\left|q|=2\beta_{0}\;\sin(\frac{\theta }{2}\right)$, where θ is the scattering angle between $k_{i}$ and $k_{s}$) is $2\beta_{0}$, covering a sphere of frequencies centered around incident spatial frequency $k_{i}$. This sphere is called Ewald’s sphere of scattering.^32^ By varying the angle of incidence of the illumination beam, different spheres centered around corresponding central frequencies can be spanned, and hence, a larger object frequency coverage can be obtained. These spheres are enclosed in another sphere called Ewald’s limiting sphere, as shown in Fig. 1(b). The maximum extent/bandwidth of spatial frequencies covered is $4\beta_{0}$.^32^

Estimation of the 3D RI distribution of an object is a well-studied inverse problem in QPI.^17^ ODT can be implemented in several ways to maximize the range of object spatial frequency coverage. The three major methods are illustrated in Figs. 1(c)–1(e). These include the rotation of the illumination beam [Fig. 1(c)],^35^ the rotation of the sample [Fig. 1(d)],^36^^,^^37^ and the axial scanning of the sample enabled by better sectioning due to the coherence gating provided by low temporal coherence and a broadband source of illumination^11^^,^^12^^,^^34^^,^^38^ [Fig. 1(e)]. In addition to these three main modes, ODT has also been implemented using illuminations with variable wavelength.^39^^–^^41^ Different reconstruction algorithms are used to reconstruct the object scattering potential, which include filtered back projection/back propagation.^24^^,^^32^^,^^42^ Two major approximations are central to ODT reconstruction: Born and Rytov.^17^ Both approximations are relevant to weakly scattering objects: the Born approximation is more suitable for samples of finer structure, whereas the Rytov approximation is more suitable for smooth objects. A detailed review of this topic can be found in Ref. 42.

The optical setup for ODT is essentially a modification of the illumination and detection paths of standard 2D QPI techniques discussed in part I of this review.^8^ Below, we discuss these modifications in different configurations; a more in-depth review of ODT can be found in Ref. 17.

#### 2.1.1 ODT with a rotating illumination

**Fig. 2:**
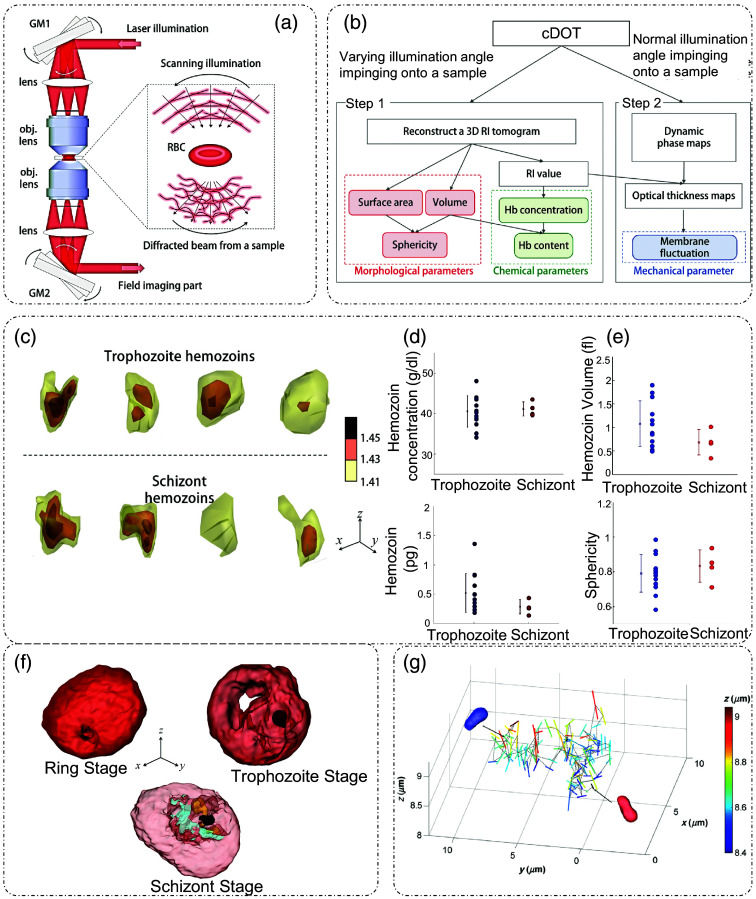
ODT by beam rotation: (a) optical setup; GM, galvano mirrors. (b) Range of possible measurements and analysis procedure schematic. Application of ODT in disease detection (here, RBCs are infected with malarial parasite) through the characterization of structural and compositional parameters of cells: (c) 3D RI distribution of hemozoins inside Pf-RBCs, (d) compositional, and (e) structural characterization of hemozoins in Pf-RBCs. (f) RI isosurface rendering of RBC infected with a malarial parasite at various stages of infection, the arrow length indicates a scale of 1  μm. (g) Real-time tracking of dynamic phenomena (here, position and orientation of a PMMA dimer), with red isosurface as the initial position and blue isosurface as the final position. (a) and (b) Reproduced with permission from Ref. 47, under CCBY-NC-SA license; (c)–(f) reproduced with permission from Ref. 48, © 2014, SPIE; and (g) reproduced with permission from Ref. 35, © 2013, Optical Society of America (Optica).

Different techniques have been employed to permit the rotation of the illumination beam, which include galvanometer-controlled mirrors,^43^ spatial light modulators,^44^^,^^45^ digital micromirror devices,^46^ etc., placed in a plane conjugate to the sample.^17^ One such example using galvano mirrors is shown in Fig. 2(a),^47^ where the illumination beam is scanned through a galvano mirror such that an oblique plane wave incidence is obtained at different angular positions. After the objective, another galvano mirror is used to descan the beam so that the beam impinges on the detection unit at a specified angle only.^43^^,^^47^ The detector unit is a DPM, which is discussed in detail in part I of this review.^8^ ODT can be used to extract several physical and chemical parameters of red blood cells (RBCs), as shown in Fig. 2(b).^47^ Using scanning illumination, the 3D RI distribution can be estimated, from which one can measure morphological information of RBCs as well as hemoglobin (Hb) concentrations that are proportional to the RI distribution.^47^

Figure 2(c) shows 3D RI tomograms of RBCs infected with malarial parasites.^48^ Hemozoin is a byproduct of Hb digestion by the parasite. Based on the 3D tomogram, the concentration of Hb in the hemozoins, volume, dry mass, and sphericity of hemozoins can be calculated, as shown in Figs. 2(d) and 2(e).^48^ Another example of a 3D RI isosurface rendering of malarial parasite-infested RBC at various stages of infection is shown in Fig. 2(f).^48^ By enabling the visualization of the 3D distribution of RI, ODT can inform the differentiation of several subcellular components inside a cell.^48^ Figure 2(g) shows a different application of ODT in which the relative positions of a PMMA dimer are tracked through time.^35^ The initial and final positions are marked by dimers of different colors, and the trajectories and orientations are marked by lines on the graph.^35^

#### 2.1.2 ODT with rotating sample

**Fig. 3:**
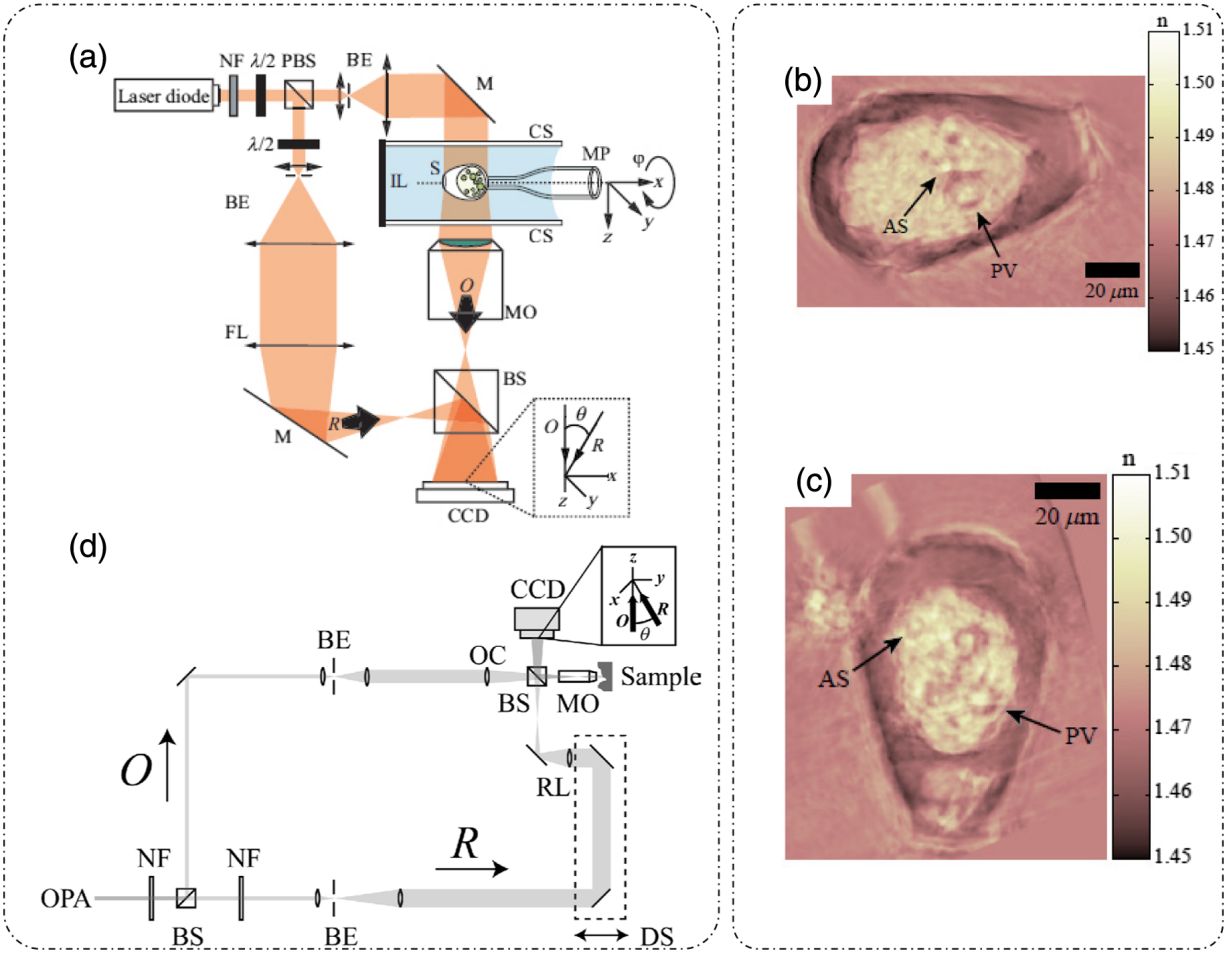
ODT by sample rotation: (a) optical setup. NF, neutral density filter; PBS, polarizing beam splitter; BE, beam expander; M, mirror; FL, field lens; BS, beam splitter; R, reference beam; IL, immersion liquid; S, sample; λ/2, half-wave plate; CS, coverslip; MP, micropipette; O, object beam; MO, microscope objective; CCD, charge-coupled device camera. (b), (c) Tomographic RI distribution of *H. papilio*. AS, algal symbionts; PV, phagocytic vacuoles. (d) ODT with wavelength scanning-optical setup. OPA, optical parametric amplifier; NF, neutral density filter; BS, beam splitter; BE, beam expander; RL, reference lens; R, reference arm; OC, object beam condenser; DS, delay system; O, object arm; MO, microscope objective; CCD, charge-coupled device camera. (a)–(c) Reproduced with permission from Ref. 37, © 2006, Optica; (d) reproduced with permission from Ref. 40, © 2006, Optica.

Instead of rotating the illumination beam, ODT can also be performed if the angular orientation of the sample is varied. Figure 3(a)^37^ shows one such experimental setup in which the sample was placed in a specially designed sample holder composed of two coverslips separated by an immersion liquid. A micropipette placed on a rotating stage was attached to a specific part of the sample and rotated through an angular span of π with a step size of 1 deg.^37^ The detection mode was transmission off-axis DHM^37^ as discussed in part I of this review.^8^ The RI distribution was recovered using a backprojection algorithm.^37^ RI tomographic reconstruction results for *Hyalosphenia papilio* (an amoeba) are shown in Figs. 3(b) and 3(c) with inner structures corresponding to algal symbionts (AS) and phagocytic vacuoles (PV) marked by arrows.^37^

#### 2.1.3 ODT with wavelength scanning

In this technique, the magnitude of the incident k-vector is varied by changing the wavelength of illumination. Figure 3(d) shows one such setup^40^ in which a variable wavelength laser [wavelength tunability is controlled through an optical parametric amplifier (OPA)] was used as an illuminating source, and holograms were recorded at 20 different wavelengths using an off-axis DHM configuration.^40^ Image reconstruction involved correcting phase aberrations using a numerical parametric lens and adjusting the size and position of the object using another numerical lens in addition to the DHM reconstruction steps outlined in part I of this review.^8^^,^^40^ An addition principle was used together with Huygens convolution formulation to sum the contribution of each wavelength.^40^^,^^41^ Using a reference plane to incorporate the same phase for all wavelengths at the respective reference plane, constructive addition takes place at the reference plane, and destructive addition happens at other planes. This results in the sectioning capability of the system, and by scanning the distance from the reference plane through the object volume, which involves the addition of an additional phase due to the additional optical path length between reference planes and planes of interest, a tomographic RI distribution can be obtained.^40^^,^^41^

#### 2.1.4 ODT with axial scanning—white-light diffraction tomography, GLIM, and Epi-GLIM

ODT can also be performed by axially scanning the specimen and using phase-shifting interferometric detection, as described in part I of this review,^8^ using a much simpler geometry such as SLIM, GLIM, or Epi-GLIM.^9^^,^^11^^,^^12^^,^^34^ Enhanced optical sectioning is obtained due to the coherence gating (that is induced by the low temporal coherence of the broadband illumination source) as well as the high numerical aperture of the imaging optics.^34^ Phase images reconstructed at different axial locations can be combined to recover the scattering potential of the object and hence the RI distribution.

**Fig. 4:**
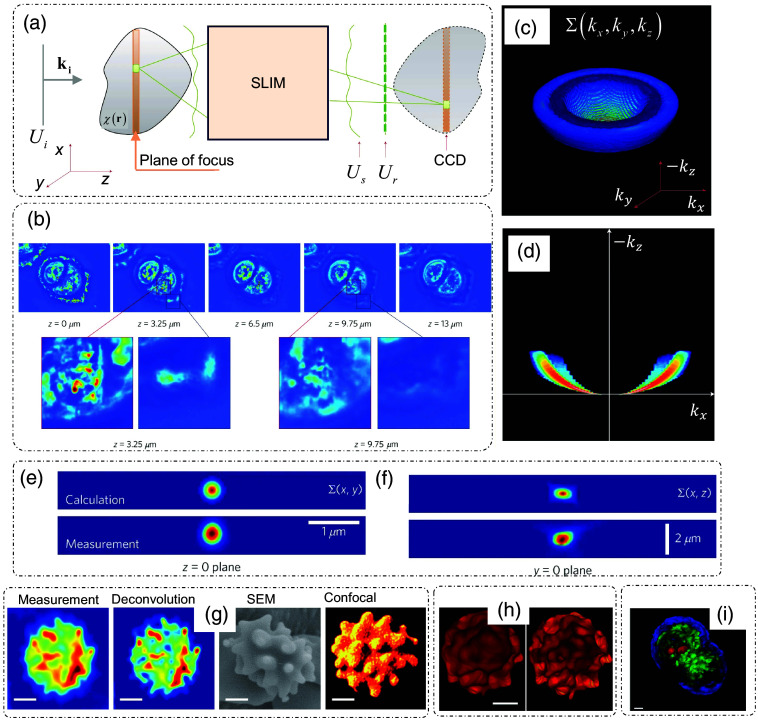
ODT by axial scanning: White-light diffraction tomography (WDT). (a) Scattering and measurement geometry; $k_{i}$, wavevector of the incident plane wave; χ(r,ω), scattering potential of the sample; $U_{i}(r,\omega )$, incident field; $U_{s}(r,\omega )$, scattered field; $U_{r}(r,\omega )$, reference field; SLIM, spatial light interference microscopy; CCD, charge-coupled device camera. (b) Sections of a single U2OS cell with different regions in-focus in different optical sections; z is the section depth, the bottom row shows the zoomed-in view of different sections, the color map represents the phase values going from blue (low) to red (high). (c) Coherent transfer function (CTF, Σ(k)) for WDT, (d) kx-kz profile of CTF, (e) xy (transverse) profile of the PSF (Σ(r)) of the WDT system, (f) xz (longitudinal/axial) profile of the PSF of the WDT system, top row: calculated, bottom row: measured for both panels (e) and (f); (g) z-slices (first two images) of Echinocyte measured through WDT before and after deconvolution and comparison with SEM and confocal images of similar cells, (h) full tomograms of echinocyte measured through WDT before and after deconvolution, (i) tomogram of an HT29 cell after deconvolution. Scale bars are 2  μm for all images in panels (g) and (h); 5  μm for panel (i). (a)–(i) Reproduced with permission from Ref. 34, © 2014, Springer Nature Limited.

White-light diffraction tomography (WDT) uses SLIM for tomographic reconstruction, as shown in Fig. 4(a).^34^ An illumination beam from a broadband source is incident on the sample, where axial scanning of the sample is performed to estimate slices of the phase distribution throughout the object at different depths. The excellent sectioning capability is shown in Fig. 4(b),^34^ where it can be seen that different parts of the cells are focused on different axial locations.

Using the theoretical framework discussed earlier in this section, the scattering potential for phase-shifting interferometric reconstruction is expressed as^34^

$$\chi (k)=\frac{\Gamma_{rs}(k;0)}{\Sigma (k)},$$ (14)

where χ(k) is the 3D Fourier transform of the object scattering potential, $\Gamma_{rs}(r,\tau )={\left\langle U_{s}(r,t)U_{r}^{*}(r,t+\tau )\right\rangle }_{t}=|\Gamma_{rs}(r,\tau )|e^{i\phi (r,\tau )}$ is the temporal cross correlation between incident and scattered fields at time delay τ, and ϕ(r,τ) is the measured phase. Using the generalized Wiener–Khinchin theorem^6^^,^^49^ Γ is expressed as $\Gamma_{rs}(r,\tau )=\int_{0}^{\infty }W_{rs}(r,\omega )e^{i\omega \tau }d\omega$. Here, $W_{rs}(r,\omega )=\langle U_{s}(r,\omega )U_{r}^{*}(r,\omega )\rangle$ is the cross-spectral density. Evaluation at τ=0 yields $\Gamma (r,\tau =0)=\int_{0}^{\infty }\langle U_{s}(r,\omega )U_{r}^{*}(r,\omega )\rangle d\omega$. The coherent transfer function of the instrument [Figs. 4(c)–4(f)] is defined as^34^

$$\Sigma (k)=\frac{1}{8{\overset{¯}{n}}^{2}}\frac{(Q^{2}+k_{⊥}^{2}{)}^{2}}{Q^{3}}S(-\frac{Q^{2}+k_{⊥}^{2}}{2Q}),$$ (15)

where $\overset{¯}{n}$ is the spatially averaged RI, S is the spectral density of the illumination source, and $Q\equiv \sqrt{\beta^{2}-k_{⊥}^{2}}-\beta$.

To recover the scattering potential of the object, a sparse deconvolution algorithm was used in conjunction with the knowledge of the measured and simulated point spread function (PSF) of the system.^34^ The results are shown in Figs. 4(g) and 4(h).^34^ Echinocytes were measured through WDT, and a slice is shown in Fig. 4(g), where measured and deconvolved phase images are shown along with scanning electron microscopy (SEM) and confocal images as ground truth for similar cells. The tomogram in Fig. 4(h) shows the spiky structure of echinocyte in both measured and deconvolved forms.^34^ Figure 4(i)^34^ shows the tomographic reconstruction of a HT29 cell after deconvolution. Different areas of the cell, such as the nucleus, cell membrane, and nucleoli, can be clearly distinguished in the tomogram.^34^

**Fig. 5:**
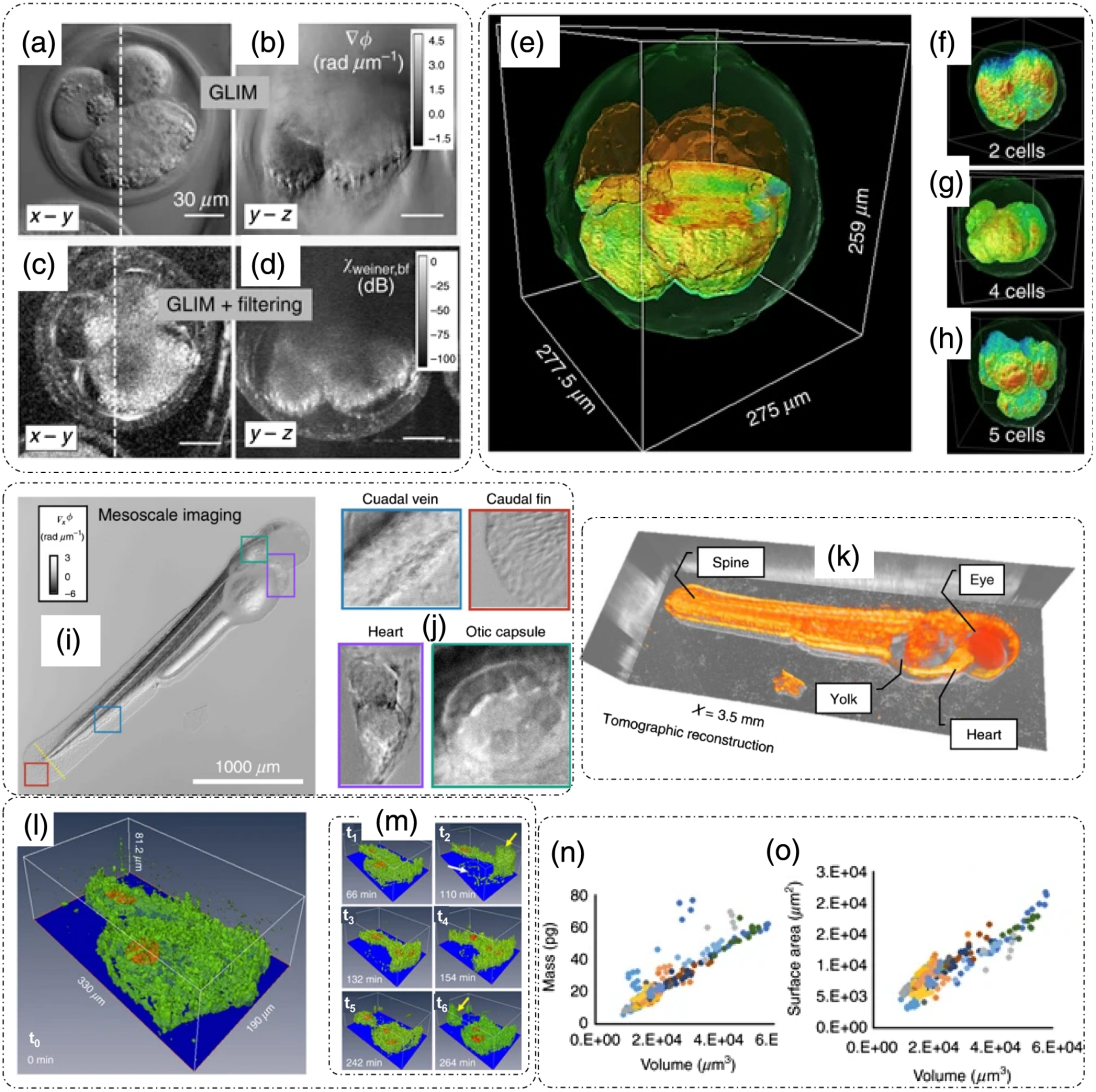
ODT by axial scanning: GLIM: (a), (c) transverse and (b), (d) longitudinal/axial views of phase gradient and filtered phase tomograms of bovine embryos, respectively. (e) Full 3D tomogram of bovine embryo, (f)–(h) tomograms of embryos at various stages of growth. Epi-GLIM: (i) live zebrafish larva quantitative phase gradient tomographic imaging, (j) distinct parts/organs of the larva, (k) tomographic reconstruction of the full larval zebrafish, (l), (m) time-lapse ($t_{0}$ to $t_{6}$) tomograms of HeLa cells measured using GLIM, with nuclei shown in orange and membranes in green. (n) Compositional and (o) structural parameters of HeLa cells can be extracted from the GLIM tomograms over time. (a)–(h) and (l)–(o) Reproduced from Ref. 11, under CC BY license; (i)–(k) reproduced from Ref. 12, under CC BY license.

A tomogram using optical phase delay measurements obtained through GLIM and Epi-GLIM is shown in Fig. 5.^11^^,^^12^ Owing to the high numerical aperture of illumination, GLIM has excellent axial sectioning capability that is used for imaging highly scattering samples, such as embryos and spheroids.^11^^,^^12^^,^^38^ To remove the background illumination artifact, GLIM images were spatially high-pass filtered before tomogram reconstruction.^11^

GLIM has also been employed to measure the growth and viability of bovine embryos.^11^ In Ref. 11, 60 bovine embryos were measured every 30 min for a week. Figures 5(a) and 5(b) show the x-y and y-z raw phase gradient profiles of the embryo,^11^ respectively, which appear after spatial filtering, as shown in Figs. 5(c) and 5(d), respectively.^11^ Using the filtered GLIM images, a tomographic reconstruction of the embryo was performed [Figs. 5(e)–5(h)] over various stages of growth.^11^ Different structural details of the embryo are clearly visible in the tomograms, namely, cells, embryo membrane, and lipid droplets.^11^

Because GLIM and Epi-GLIM are widefield methods, fast imaging of large structures such as a zebrafish larva is also possible.^12^ Imaging a live zebrafish larva with Epi-GLIM as shown in Figs. 5(i)–5(k) was reported in Ref. 12. Distinct parts/organs of the organism that include the heart, fins, otic capsule, and caudal vein can be detected [Fig. 5(j)].^12^ A tomographic reconstruction from axially scanned and spatially high-pass filtered phase images of the whole organism is shown in Fig. 5(k).^12^ Figures 5(l) and 5(m) show the 3D phase tomograms of HeLa cells measured over 7.7 h with arrows indicating the changes in cells during mitosis.^11^ Different quantitative markers can be extracted from such tomographic measurements, as shown in Figs. 5(n) and 5(o).^11^ The dry mass of cells measured during a 21-h period is plotted with respect to the volume of the cells in Fig. 5(n), which shows a linear dependency.^11^ Similarly, the surface area of cells also varied linearly with the volume, as shown in Fig. 5(o).^11^

Soto et al.^50^ reported the development of a partially coherent ODT (PC-ODT) technique that relies on intensity measurements for extracting the 3D RI distribution of samples. PC-ODT employs a partially coherent brightfield illumination coupled with a fast electronically programmable focus tunable lens to acquire a 3D stack of brightfield intensity images. This stack is represented in the spatial-frequency domain as^50^

I˜(k)=I0δ(k)+Re{χ˜(k)}HP(k)+Im{χ˜(k)}HA(k), (16)

where $I_{0}$ is the background intensity and $H_{P}(k)$ and $H_{A}(k)$ are the phase and absorption optical transfer functions, respectively; a detailed description of these terms can be found in Ref. 50. Soto et al.^50^ assumed that the imaginary part of the scattering potential (responsible for absorption) is related to the real part of the scattering potential (responsible for scattering) as Im{χ(r)}=ξ Re{χ(r)}, implying that the sample is weakly absorbing. Under this assumption, using Eq. (16) in conjunction with Weiner deconvolution yields the solution for scattering potential in the **k**-domain as^50^

Re{χ˜(k)}=(I(k)−I0δ(k))(Hp(k)+ξHA(k))*|Hp(k)+ξHA(k)|2+β, (17)

where β is the regularization parameter. PC-ODT is particularly useful for the simpler optical setup involved and carries the benefits of low-coherence microscopy, such as the absence of speckle noise with better depth sectioning.^50^

Deconvolutions are prone to artifacts in the reconstruction. An improvement over PC-ODT in terms of reduced artifacts in the reconstructed RI distribution was presented by Hugonnet et al.^51^ with a technique called plural efficient patterns for self-interfering ODT.^51^ The study introduces a simulated annealing-based optimization procedure (which involved expressing illumination pupil intensity as a linear sum of Zernike polynomials and aimed at increasing the signal-to-noise ratio (SNR) of the phase transfer function, with noise assumed to be Gaussian white noise) to get optimal illumination patterns for deconvolution phase microscopy that leads to a homogenous sampling of spatial frequencies.^51^

In all of the ODT techniques, multiple measurements are essential to extract full 3D object information. However, this aspect also degrades the speed of operation, thereby limiting the ODT techniques to static or slowly varying temporal observations. Li et al.^52^ proposed an annular illumination intensity diffraction tomography (aIDT) to improve the temporal resolution. The optical setup for aIDT involves annular LED illumination combined with a standard brightfield microscope.^52^ The height of the LED illumination source is determined such that the NA of illumination matches the NA of the objective for maximal frequency space coverage.^52^ Acquisition involves illuminating the sample with a small set of angular illuminations (8 to 24). Reconstruction involves slice-wise 3D deconvolution with Tikhonov regularization of the measurement with the corresponding optical transfer functions.^52^ A self-calibration method is also presented by Li et al.^52^ to improve the RI reconstruction for out-of-focus slices. The major significance of aIDT was demonstrated by a dynamic 3D RI measurement of *Caenorhabditis elegans* *in vitro* at a volume rate of 10.6 Hz.^52^

Applications of ODT include single cell-level studies of the pathophysiological effects of infection and diseases for cells such as RBCs, white blood cells, cancer cells, and virus-infected cells.^17^ ODT can be inherently slow due to the multiple scanning steps involved. However, a real-time reconstruction of the RI distribution of the sample using sparse angle illumination and performing the computations on a graphics processing unit was reported in Ref. 35. The reported reconstruction time was 0.21 s for a volume of 96×96×96  voxels.^35^

#### 2.1.5 Other ODT configurations—hyperspectral ODT (HS-ODT), darkfield-ODT, and ODT for measuring birefringent samples

**Fig. 6:**
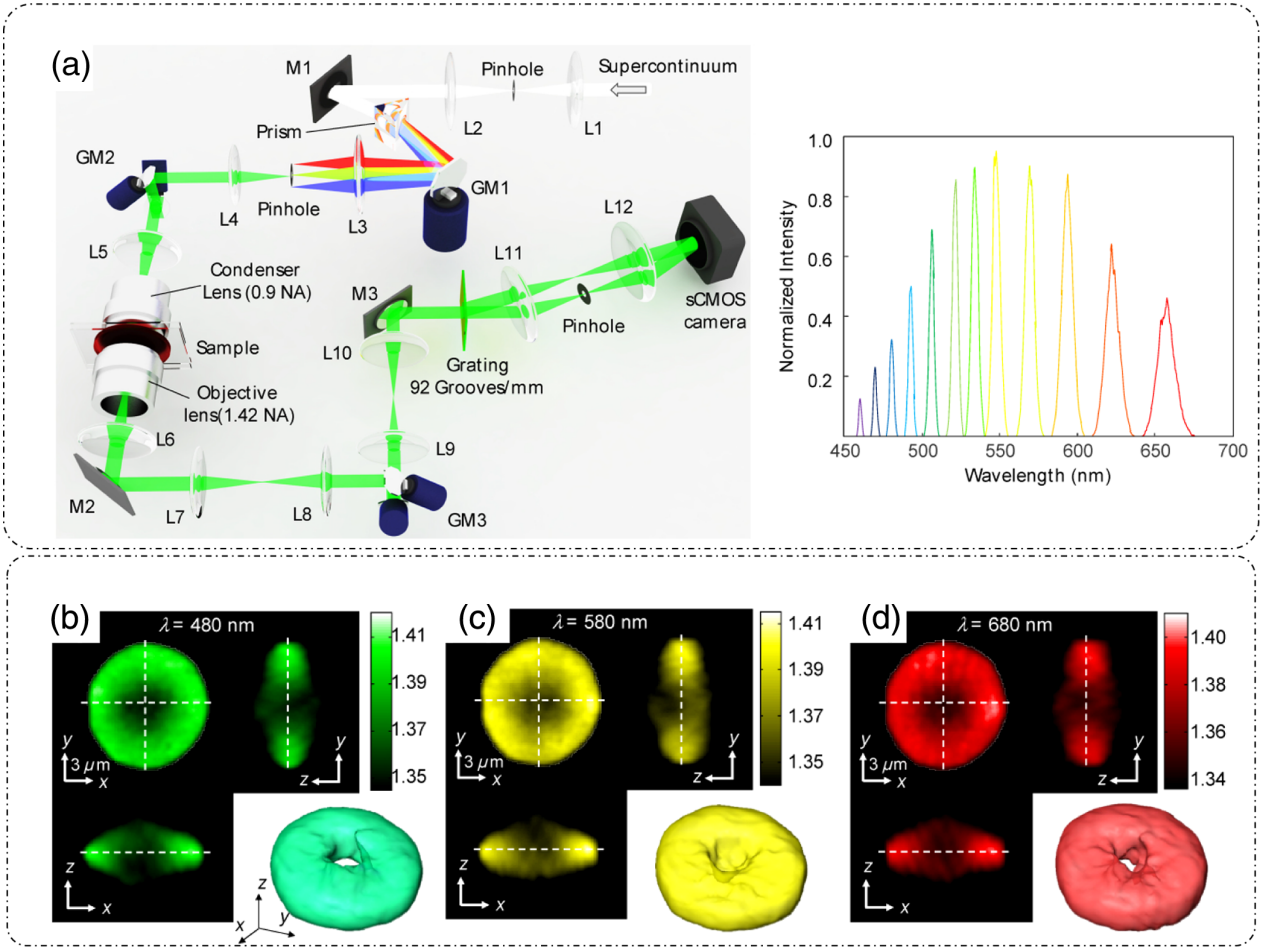
HS-ODT. (a) Optical setup (left); M, mirrors; GM, galvano mirrors; L, lens; and illumination spectrum (right) and (b)–(d) 3D RI tomograms of an RBC at three different wavelengths. (a)–(d) Reproduced with permission from Ref. 53, © 2016, Optica.

Other configurations of ODT exist in the literature. One such example is hyperspectral ODT (HS-ODT),^53^ as shown in Fig. 6(a), where a broadband source is dispersed into constituent wavelengths and ODT imaging is performed for each wavelength to decouple the effect of wavelength dependence RI variations. The hyperspectral tomograms of RBCs measured at three different wavelengths are shown in Figs. 6(b)–6(d), all showing slightly different characteristic RI distributions.^53^

**Fig. 7:**
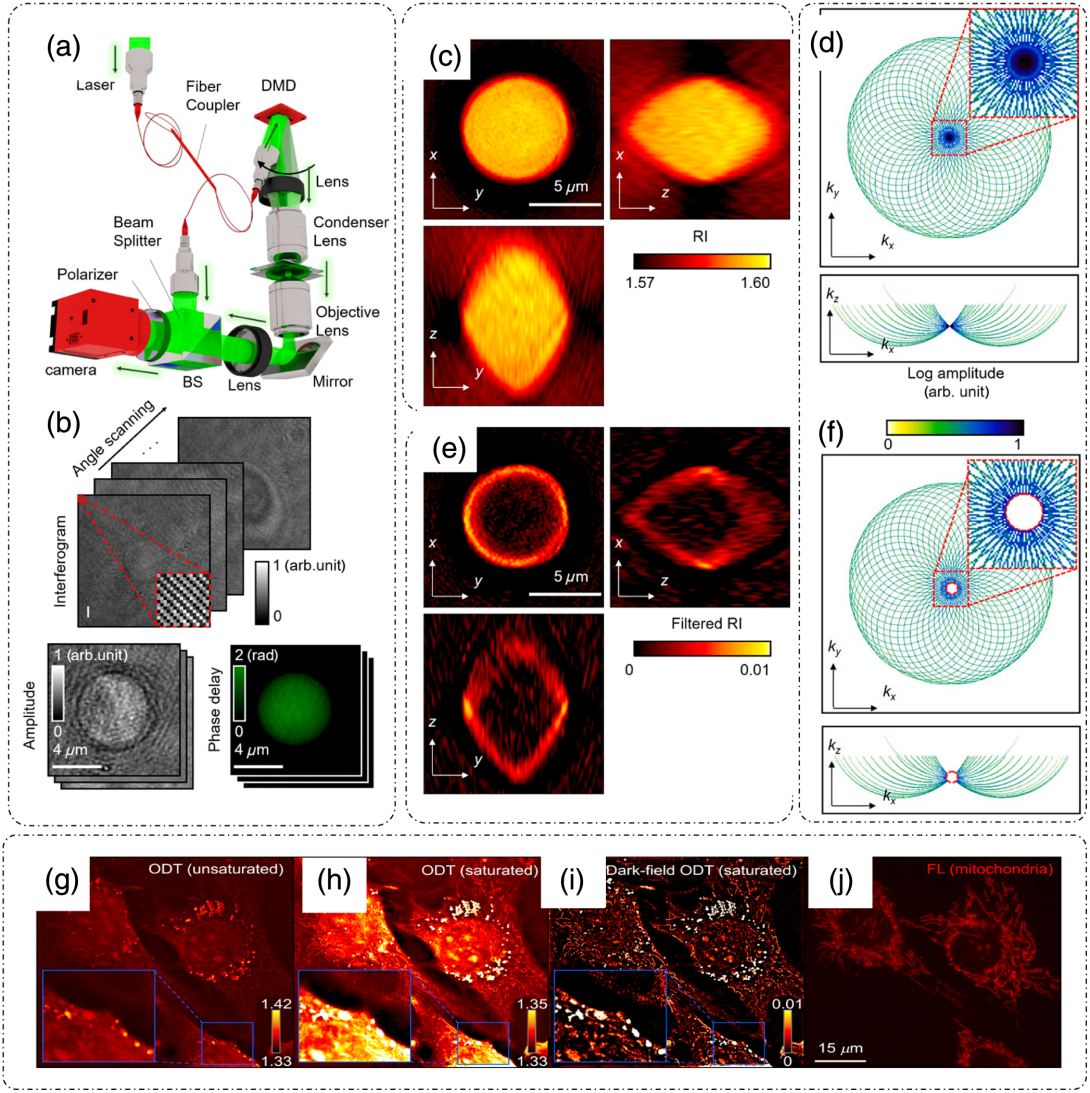
Computational dark-field ODT: (a) setup; BS, beam splitter; DMD, digital micromirror device. (b) Amplitude and phase reconstruction of a polystyrene microsphere from multiple interferograms recorded at different angles. (c) RI distribution of the polystyrene microsphere for a normal ODT. (d) Ewald sphere showing frequency coverage of the normal ODT. (e) RI distribution after high-pass filtering (c), (f) Ewald sphere showing frequency coverage for panel (e). Cross-sectional images of tomograms of an NIH-3T3 cell for: (g) conventional ODT and (h) contrast-adjusted version of panel (g). (i) Computational dark-field ODT of the same field of view as in panel (g). (j) Mitochondrial fluorescence image of the same field of view for comparison. (a)–(j) Reproduced from Ref. 54, under CC BY license.

Efforts have been made to increase the contrast and resolution of ODT. In Ref. 54, spatial high-pass filtering in the 3D Fourier space of ODT images was performed, which enhanced the contrast significantly. Chang et al.^54^ referred to this technique as dark-field ODT. Figure 7(a) shows a common ODT setup with varying angular illumination using a DMD, as discussed previously.^54^ There is no change in the optical setup of ODT to produce dark-field ODT images because the enhancement is achieved through computation.^54^ Figure 7(b) shows the ODT reconstruction process in which a complex field for a polystyrene bead is recovered from the hologram at each illumination angle.^54^ The RI distribution of the polystyrene bead for normal ODT is shown in Fig. 7(c), and the corresponding frequency coverage in the Ewald sphere is shown in Fig. 7(d).^54^ After applying a spatial high-pass filter to reject the low-frequency background, as evident by the central hole of the Ewald sphere in Fig. 7(f), the RI distribution for the bead approaches dark-field-like images [Fig. 7(e)].^54^ The effect of contrast improvement is more pronounced in biological samples with underlying cell organelle distributions. Figure 7(g) shows a conventional ODT study of an NIH-3T3 cell.^54^ The image is contrast-adjusted to show the mitochondria in Fig. 7(h).^54^ The enhancement in contrast due to dark-field ODT is evident in Fig. 7(i), which can be compared with the fluorescence image of the same cells in Fig. 7(j).^54^

The various ODT configurations discussed so far were based on scalar wave equations. Such configurations did not involve the polarization of the electric field when estimating the 3D RI distribution. Such an exclusion is justified for isotropic (non-birefringent) samples; however, for birefringent samples, a vector formalism of the scattering theory is an appropriate method for determining the scattering potential and hence the 3D RI distribution of such samples.

**Fig. 8:**
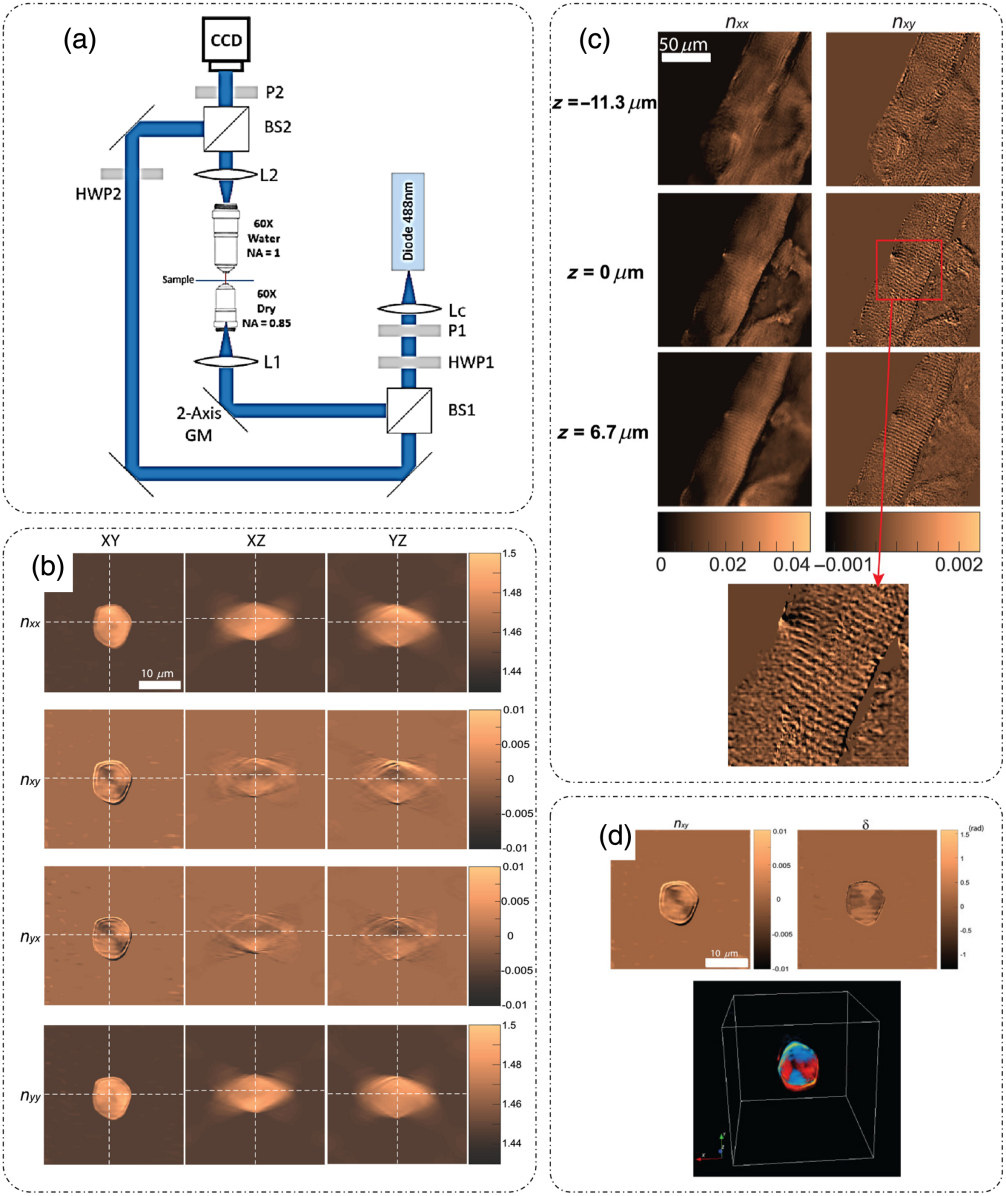
PS-ODT. (a) Optical setup; L, lens; HWP, half-wave plate; GM, galvano mirror; BS, beam splitter; P, polarizer; CCD, charge-coupled device camera. (b) 3D RI tensor reconstruction of a cornstarch granule. (c) XY profiles at three depths of 3D RI tensor components for a 20  μm thick muscle tissue, with the inset showing a 2.5× zoomed-in view of the red box in the $n_{xy}$ map in a z=0  μm slice, clearly showing the striated structure. (d) XY profiles of 3D reconstruction of (top left) $n_{xy}$ and (top right) phase retardation with a 3D view of the phase retardation at the bottom for cornstarch granule. (a)–(d) Reproduced with permission from Ref. 55, © 2021, Optica.

Saba et al.^55^ introduced polarization-sensitive ODT (PS-ODT) for characterizing anisotropic samples. The optical setup is shown in Fig. 8(a).^55^ It is an off-axis holographic setup that involves the rotation of illumination beams. The major difference here is the use of a pair of +45 and −45 deg (from the X direction) polarized illumination beams with the holographic measurements conducted in the XY plane for each illumination angle. The choice of the specific polarization states was justified to avoid random phase values in the background.^55^ Light from the diode laser (488 nm) is collimated by the lens (Lc), and the state of polarization is controlled by P1 (polarizer) and HWP1 (half-wave plate). The beam is split into a sample and a reference beam by a beam splitter (BS1). The selected illumination polarizations are +45 and −45  deg. The illumination is angularly scanned using two-axis galvano mirrors. Lens L1 and objective lens (60 × dry NA = 0.85) (in a 4F configuration) illuminate the sample, and the 60× water objective and lens L2 (another 4F configuration) collect the light from the sample and project it to the charge-coupled device camera (CCD) plane. The polarization state of the reference beam is such that it is at 45 deg with respect to the X/Y axes. The analyzer (P2) is used to select the polarization of the sample beam after combining the sample and reference beams by another beam splitter (BS2).^55^

Below, we discuss the mathematical formulation of PS-ODT as introduced in Ref. 55.

The vectorial wave equation describing a monochromatic light–matter interaction upon propagation through a weakly scattering anisotropic object surrounded by an isotropic medium is expressed as^55^

$$(\nabla^{2}+\beta_{0}^{2}{\overset{↔}{\varepsilon }}_{r})E(r)=0,$$ (18)

where E is the electric field vector, ${\overset{↔}{\varepsilon }}_{r}=n_{0}^{2}I_{3}+2n_{0}{\overset{↔}{\delta }}_{n}=n_{0}^{2}I_{3}+{\overset{↔}{\delta }}_{\varepsilon }$ is the relative permittivity tensor, $I_{3}$ is an identity matrix, ${\overset{↔}{\delta }}_{n}$ is the RI tensor of the object, ${\overset{↔}{\delta }}_{\varepsilon }$ is the permittivity tensor of the object, both defined with respect to the surrounding media, and $n_{0}$ is the RI of the media. The remaining symbols carry the same meaning as previously defined in this paper. Equation (18) is further expressed as the vectorial inhomogeneous Helmholtz equation:^55^

$$(\nabla^{2}+\beta_{0}^{2}n_{0}^{2})E(r)=-\overset{↔}{\chi }(r)E(r),$$ (19)

where $\overset{↔}{\chi }(r)=\beta_{0}^{2}{\overset{↔}{\delta }}_{\varepsilon }=\beta_{0}^{2}({\overset{↔}{\varepsilon }}_{r}-n_{0}^{2})$ is the object’s scattering potential tensor. Upon substituting $E(r)=E_{S}(r)+E_{i}(r)$, meaning that the total field is the sum of the scattered and incident fields, and applying the first-order Born approximation (as discussed previously in Sec. 2), Eq. (19) simplifies to^55^

$$(\nabla^{2}+\beta_{0}^{2}n_{0}^{2})E_{s}(r)=-\beta_{0}^{2}{\overset{↔}{\delta }}_{\varepsilon }E_{i}(r)=-\overset{↔}{\chi }(r)E_{i}(r).$$ (20)

The solution to Eq. (20) is expressed as^55^

$$E_{s}(r)=\int \overset{↔}{G}(r,r^{\prime })\overset{↔}{\chi }(r^{\prime })E_{i}(r^{\prime })d^{3}r^{\prime },$$ (21)

where $\overset{↔}{G}(r,r^{\prime })$ is the Green’s function tensor defined as $[e^{i\beta_{0}n_{0}(r-r^{\prime })}/|r-r^{\prime }|]I_{3}$.

Saba et al.^55^ simplified the above formalism by neglecting the z component of the tensors and hence reducing them to 2×2 tensors. The reason behind this simplification is that there can be only two independent polarization components perpendicular to the axis of propagation of the incident light per illumination angle.^55^

Considering a plane-wave illumination with incident wavevector $k_{i}$, $E_{i}(r)=E_{i}e^{ik_{i}.r^{\prime }}$, utilizing a Jones matrix formulation, and following the mathematics as explained in Sec. 2, the scattering potential is expressed in the spatial frequency domain as^55^

$$\overset{↔}{\overset{˜}{\chi }}(k_{x}-k_{xi},k_{y}-k_{yi},\gamma -k_{zi})=-2i\gamma {\mathrm{FT}}_{2D}\{[\begin{array}{cc} E_{sx1} & E_{sx2}\\ E_{sy1} & E_{sy2} \end{array}]{\left[\begin{array}{cc} E_{ix1} & E_{ix2}\\ E_{iy1} & E_{iy2} \end{array}\right]}^{-1}\}(k_{x},k_{y}),$$ (22)

where $\gamma =\sqrt{\beta_{0}^{2}n_{0}^{2}-k_{x}^{2}-k_{y}^{2}}$. Here, the subscripts 1 and 2 of each matrix element represent the two polarization states of the incident illumination beam. It is to be noted that Eq. (22) differs in a multiplicative constant from the original Ref. 55 as we have modified the description of $\overset{↔}{\chi }$ to exclude the 4π term for maintaining consistency within our paper. Under the Rytov approximation (first order), the total field is expressed as $E(r)=E_{i}(r)e^{\varphi }$, where φ is a complex exponent. Hence, the complex phase tensor $\overset{↔}{\varphi }=\mathrm{logm}(\frac{E(r)}{E_{i}(r)})$ is used in the inverse-scattering formulation (as the term $e^{ik_{i}.r}\overset{↔}{\varphi }$) to estimate $\overset{↔}{\chi }$ and subsequently the object’s RI tensor ${\overset{↔}{\delta }}_{n}$ as described in detail in Ref. 55. The authors of Ref. 55 used an iterative denoising algorithm utilizing a 3D total variation (TV) regularizer for reconstruction.

To obtain a quantitative measure of birefringence, Saba et al.^55^ performed an eigen-value decomposition analysis of the RI tensor. Phase retardation ψ upon propagation through a small distance dz was determined to be proportional to the difference between the eigen-values of the RI tensor $\mu_{n}^{1,2}$ as $\psi =\beta_{0}dz(\mu_{n}^{1}-\mu_{n}^{2})$. They were also able to quantify the directionality of the sample’s slow/fast axis by analyzing the orientation of the eigenvector.^55^

The results of PS-ODT are shown in Fig. 8.^55^ Figure 8(b) shows the XY, XZ, and YZ profiles of the 3D RI tensor of a cornstarch granule (a birefringent sample).^55^ The XY profiles of the $n_{xx}$ and $n_{xy}$ components of the 3D RI tensor for a mouse muscle tissue (a birefringent sample) slice (20  μm) at different depths within the sample are shown in Fig. 8(c),^55^ where the striated structure is much more visible in the $n_{xy}$ component. The $n_{xy}$ component and the resultant phase retardation map for the cornstarch granule are shown in Fig. 8(d), which shows the quantitative nature of the measurements for characterizing the birefringence of the sample.^55^

**Fig. 9:**
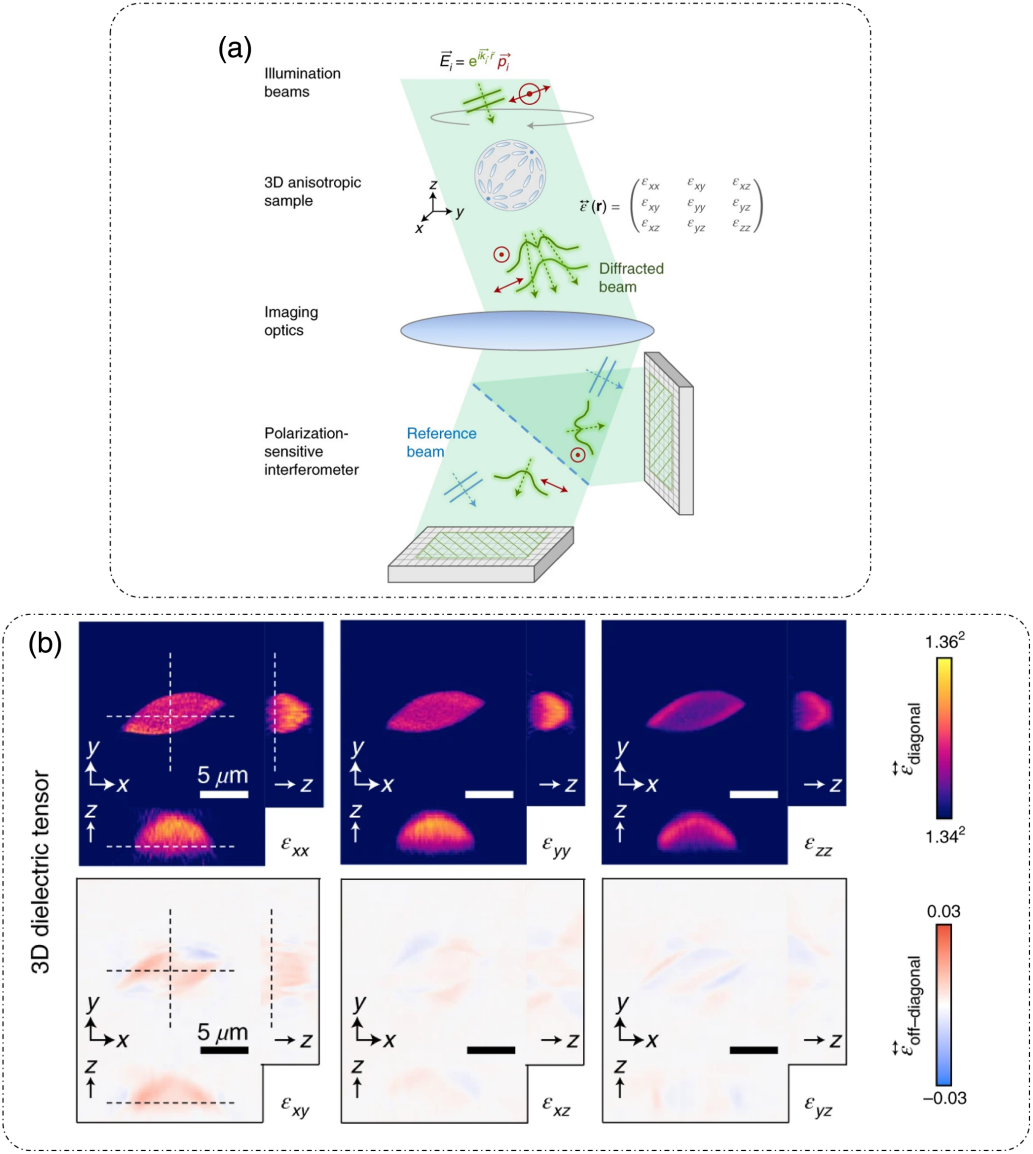
DTT: (a) optical schematic; $E_{i}$, i’th angular incident electric field vector with polarization vector $p_{i}$ and wavevector $k_{i}$. (b) 3D dielectric tensor $\overset{↔}{\varepsilon }(r)$ reconstruction of an anisotropic sample. (a) and (b) Reproduced with permission from Ref. 56, © 2022, The Authors, under exclusive license to Springer Nature Limited.

PS-ODT^55^ involves a simplification in which one component (along z direction) of the respective tensors is omitted to arrive at the inverse scattering solution. Dielectric tensor tomography (DTT)^56^ is another polarization sensitive DT method, in which a solution that utilizes the Fourier differentiation theorem is provided to avoid such an omission. It is a polarization sensitive off-axis holographic system, as shown in Fig. 9(a),^56^ in which multiple plane-wave illumination beams with specific polarizations and angular orientation are used to illuminate the sample. The light after the sample interferes with an off-axis reference beam and is recorded in two orthogonal polarization states using a pair of linear analyzers placed before the image sensors.^56^ DTT is based on ODT under Rytov’s approximation, which involves approximating the total field as $E(r)=E_{i}(r)e^{\varphi }$, as discussed above.^56^

Below, we provide a brief discussion of the mathematical formalism introduced by Shin et al.^56^ Starting from the inhomogeneous vectorial Helmholtz equation for an anisotropic sample given in Eq. (19), applying Rytov’s approximation, and substituting $\varphi e^{\varphi^{0}}=\varphi e^{ik_{i}.r}$ (as detailed in the Supplementary information of Ref. 56) yields the inverse scattering solution:^56^

$$\overset{↔}{\overset{˜}{\chi }}(k_{x}-k_{xi},k_{y}-k_{yi},\gamma -k_{zi})p_{i}=-2i\gamma e^{-i\gamma z}p_{i}\circ \overset{˜}{\varphi }(k_{x}-k_{xi},k_{y}-k_{yi};z),$$ (23)

where p indicates the input polarization state of the illumination.^56^

Shin et al.^56^ utilized the concept of the Fourier differentiation theorem to expand the slightly shifted scattering potential in the k-space as^56^

$$\overset{↔}{\overset{˜}{\chi }}(k+\Delta k)={\mathrm{FT}}_{3D}\{(1-ir\cdot \Delta k)\overset{↔}{\chi }(r)\}.$$ (24)

The slightly tilted illumination results in the introduction of an orthogonal component with respect to the original polarization. The effect of the tilt is expressed as^56^

$$\overset{↔}{\overset{˜}{\chi }}(k+\Delta k)p_{i}=-2i\gamma e^{-i\gamma z}(p_{i}+\Delta p_{i})\circ \overset{˜}{\varphi }(k_{x}-k_{xi}-\Delta k_{xi},k_{y}-k_{yi}-\Delta k_{yi};z).$$ (25)

Reverting to spatial coordinates and using Eq. (24) results in^56^

$$(1-ir\cdot \Delta k)\overset{↔}{\chi }(r)p_{3}={\mathrm{IFT}}_{3D}[-2i\gamma e^{-i\gamma z}p_{3}\circ \overset{˜}{\varphi }(k_{x}-k_{xi}-\Delta k_{xi},k_{y}-k_{yi}-\Delta k_{yi};z)].$$ (26)

The final scattering potential tensor is thus expressed as^56^

$$\overset{↔}{\chi }(r)=[\begin{array}{c} {\mathrm{IFT}}_{3D}(-2i\gamma e^{-i\gamma z}p_{1}\circ {\overset{˜}{\varphi }}_{1})\\ {\mathrm{IFT}}_{3D}(-2i\gamma e^{-i\gamma z}p_{2}\circ {\overset{˜}{\varphi }}_{2})\\ {\mathrm{IFT}}_{3D}(-2i\gamma e^{-i\gamma z}p_{3}\circ {\overset{˜}{\varphi }}_{3}) \end{array}]{\left[\begin{array}{c} p_{1}\\ p_{2}\\ (1-ir.\Delta k)p_{3} \end{array}\right]}^{-1},$$ (27)

where $p_{3}$ is the slightly tilted illumination polarization and ${\overset{˜}{\varphi }}_{3}=\overset{˜}{\varphi }(k_{x}-k_{xi}-\Delta k_{xi},k_{y}-k_{yi}-\Delta k_{yi};z)$. Shin et al.^56^ employed singular value decomposition on the reconstructed dielectric tensor ${\overset{↔}{\varepsilon }}_{r}$ for extracting the principal refractive indices and the 3D director distributions.^56^ Figure 9(b) shows the complete characterization of a 3D dielectric tensor for an anisotropic sample, reconstructed using DTT.^56^

**Fig. 10:**
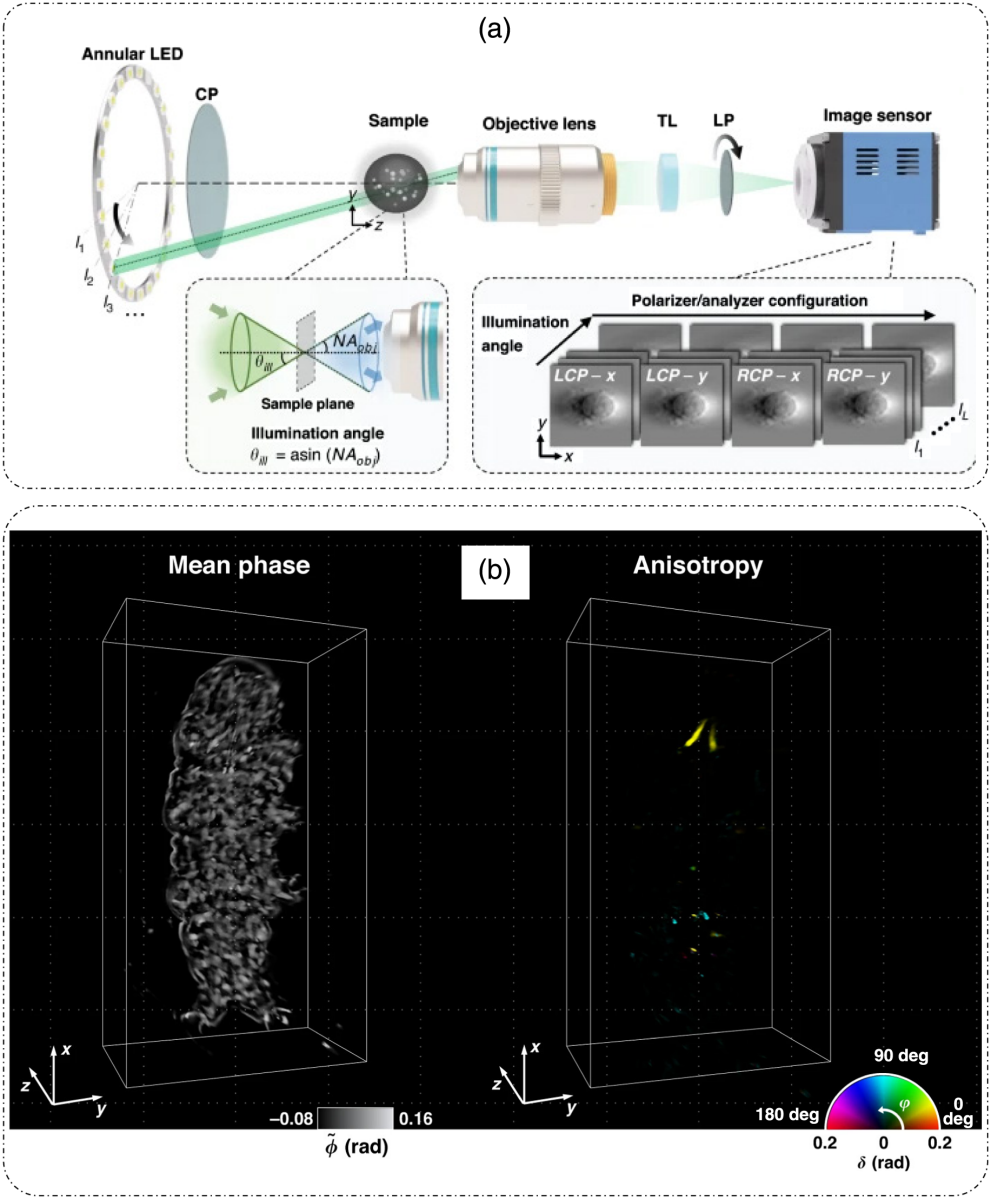
PS-IDT. (a) Optical setup and acquisition design; CP, circular polarizer; TL, tube lens, LP, linear polarizer; $l_{i}$, i’th LED/angular illumination; LCP, left circular polarization; RCP, right circular polarization. (b) 3D mean phase map and 3D anisotropy map of a full tardigrade over a volume of 150  μm×80  μm×60  μm. (a) and (b) Reproduced from Ref. 57, under CC BY license.

The above-mentioned polarization sensitive ODT techniques are based on single (weak)-scattering assumptions and employ interferometric detections. Song et al.^57^ introduced a polarization-sensitive intensity diffraction tomography (PS-IDT) without the weak scattering assumptions, extending the usability of the technique to characterize the Jones matrix of highly scattering, anisotropic samples.^57^ Because the method is based on intensity measurements, the optical setup is much simpler and involves an annular LED illumination in a standard polarization-mode brightfield microscope, as shown in Fig. 10(a).^57^ The sample is illuminated by the annular plane wave illumination (with the incident angle matched to the NA of the objective to ensure optimal spatial-frequency coverage) by sequentially switching on each LED, such that a set of oblique illuminations is produced. The polarization of each illumination is controlled using a rotating wheel that houses circular polarizers for switching between left- and right-handed circular polarization states. The light scattered from the sample is collected by the objective lens and projected onto the image sensor (scientific complementary metal-oxide semiconductor camera) by the tube lens. The polarizer just before the camera is used to select between the two orthogonal polarization states for measurement. The imaging involves the acquisition of 24 intensity images per polarizer–analyzer configuration [four configurations as shown in Fig. 10(a)], totaling 96 intensity images per experiment.^57^ The reconstruction principle now relies on intensity measurements. The forward model is based on a vectorial multi-slice beam propagation model, which involves a sequential slice-by-slice propagation of electric field through a 3D sample utilizing an angular spectrum propagator and Jones matrix formulation.^57^ The inverse problem for the reconstruction of the Jones matrix was formulated as the minimization of the $l_{2}$ norm between the square root of intensity measurements and forward model estimates with a 3D TV regularizer.^57^ Song et al.^57^ also performed eigenvalue decomposition of the Jones matrix for quantification of linear retardance and optic axis directions. Figure 10(b) shows the 3D mean phase map and anisotropy map for an entire tardigrade sample reconstructed by PS-IDT.^57^

Recently, Ge et al.^58^ introduced single-frame label-free cell tomography (SILACT). It combines off-axis DHM with machine learning (a pretrained, physics-incorporating deep neural network) to enhance the acquisition speed for 3D RI measurements using only one multiplexed angular illumination (four angle illuminations are multiplexed by overlapping Lee holograms on a DMD) and a high-speed camera.^58^ The speed of 3D RI acquisitions was reported to be greater than 10,000 volumes/s.^58^ For imaging cells in a microfluidic channel in a cytometer-like mode, SILACT’s acquisition speed was demonstrated to be 20,000 cells/s.^58^ Ge et al.^58^ also demonstrated the capability of SILACT to measure shear-induced deformations in RBCs at the sub-millisecond temporal scale.

All of the 3D tomographic techniques suffer from the missing cone problem, which is due to the limitation of the illumination angle and the imaging optics being unable to probe the entire range of spatial frequencies of the object. Solutions based on conventional signal processing algorithms and modern machine learning approaches can be found in the literature.^59^

### 2.2 Phase-Resolved Optical Coherence Tomography

**Fig. 11:**
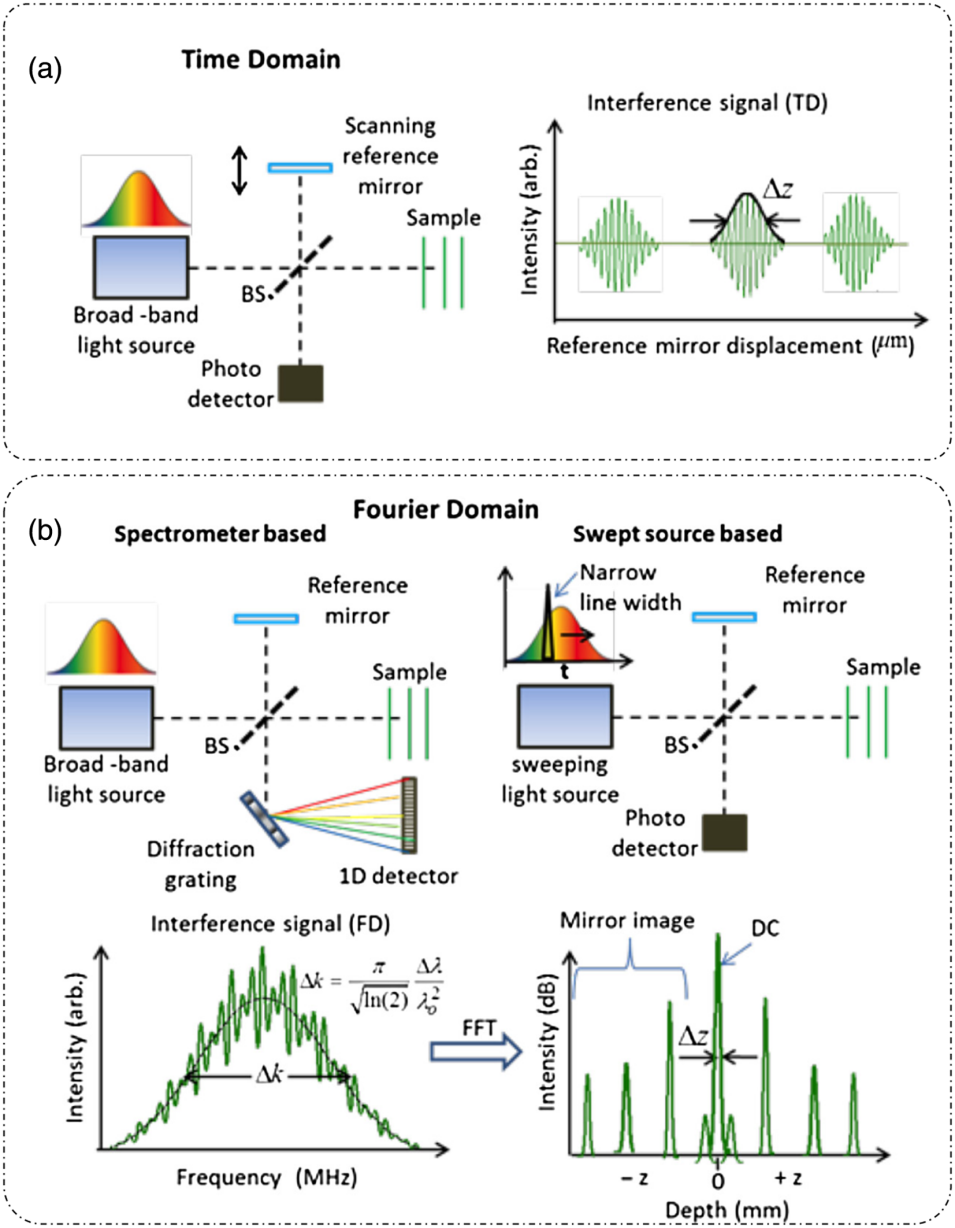
Optical schematic for (a) TD-OCT system and (b) frequency-domain OCT system.(a) and (b) Reproduced from Ref. 62, under CC BY license.

Optical coherence tomography (OCT) is a label-free, intrinsic contrast, low-coherence interferometric technique based on back-scattered light that has seen widespread clinical adoption for retinal imaging.^60^^,^^61^ The basic optical setup of a time-domain OCT (TD-OCT) is shown in Fig. 11(a).^62^ Light from a low-temporal coherence optical source (e.g., a Ti-sapphire laser) is divided into a reference beam and an object beam. Both of these beams travel separate arms of a Michelson interferometer. The reference beam is scanned temporally using a mirror in the reference path, and the sample beam goes through different planes of focus in the sample. At each focal plane within the sample, the reference mirror is scanned axially to produce the depth profile at that plane, also called an amplitude scan or A-scan. The intensity of the detector at a particular spatial location is written as^6^

Id(τ)=IR+Is+2 Re[ΓRS(τ)], (28)

where $I_{R}$ is the intensity of the reference field $U_{R}(t+\tau )$, $I_{S}$ is the intensity of the backscattered field from sample $U_{S}(t)$, and $\Gamma_{RS}(\tau )=\langle U_{R}(t+\tau ).U_{s}^{*}(t)\rangle$ is the cross-correlation between the sample field and the reference field at the temporal delay $\tau =\frac{2\Delta Ln}{c}$ (at the scanning mirror displacement of ΔL in a medium of RI n). The magnitude of the cross-correlation $\left|\Gamma_{RS}(\tau )\right|$ is the A-scan measurement in TD-OCT.

To obtain a transverse image of the whole sample, the sample beam is translated in the transverse direction to get a brightness scan or B-scan. The low temporal coherence of the light source helps in optical sectioning by coherence gating. Coherence gating is a phenomenon caused by the narrow range of coherency between the two interfering beams, such that the interference will be observed within the coherence length of the source ($l_{c}$), nearly on the order of a few micrometers for low-coherence, broadband sources, outside of which the beams become incoherent to each other and interference contrast washes out. The microstructural information inside the sample is estimated by extracting the depth information from the recorded intensity. Thus, the free-space axial resolution of OCT is $\Delta z=(2\;\ln\;2/\pi )\frac{\lambda_{0}^{2}}{\Delta \lambda }\propto l_{c}$, where $\lambda_{0}$ is the central wavelength of the broadband source with spectral bandwidth Δλ.^60^ Hence, the axial resolution is only dependent on the coherence length $l_{c}$ of the source and not on the optics of the system, whereas the transverse resolution is still limited by the NA of the optics.^6^ OCT achieves a transverse resolution ranging from 15 to 1  μm, and the achievable imaging depth is up to 3 mm in tissues excluding the eye, where much higher imaging depths are achieved due to the lower scattering of light.^60^

There are many variants of OCT that improve on the traditional TD-OCT in terms of resolution and speed of acquisition.^18^ The temporal scanning of the reference mirror is replaced by a single-shot frequency-domain measurement in Fourier-domain OCT (FD-OCT). Hence, instead of recording autocorrelation, a spectral density measurement is made, from which A-scan is obtained after a Fourier transform, thereby improving the acquisition speed, which is limited only by the detection optics. In an FD-OCT system, Fourier-domain measurements are made by replacing the photodetector with a spectrometer (grating with a line detector) in a technique named spectral-domain OCT (SD-OCT) [Fig. 11(b)]^62^ or using a swept source with a photodetector, where the source wavelength is scanned over a broad band, temporally at high speeds resulting in another technique called swept-source OCT [Fig. 11(b)].^18^^,^^62^

The measured spectral signal at the detector is^6^

$$S(\omega )=S_{R}(\omega )+S_{S}(\omega )+2\sqrt{S_{R}(\omega )S_{S}(\omega )}\;\cos[\phi_{0}(\omega )+\phi (\omega )],$$ (29)

where $S_{R}(\omega )$ is the unmodified spectrum of the source, $S_{S}(\omega )$ is the source spectrum modified by the sample, $\phi_{0}(\omega )$ is the phase difference due to the fixed path length mismatch between the two arms of the interferometer, and ϕ(ω) is the spectral phase perturbation introduced by the sample. The third term in Eq. (29) is the cross-spectral density $W_{RS}(\omega )=U_{R}(\omega )U_{S}^{*}(\omega )$. A-scan information can be retrieved from the cross-correlation, which in turn is then computed from the cross-spectral density measurement using the generalized Wiener–Khinchin theorem as^6^^,^^49^

$$\Gamma_{RS}(\tau )=\int W_{RS}(\omega )e^{-i\omega \tau }d\omega$$ (30)

Here, we note that a complete complex analytic signal

$$\Gamma_{RS}(\tau )=A(\tau )e^{i\phi (\tau )}$$ (31)

is recovered, providing both amplitude (A) and phase (ϕ) information. Numerous informative reviews on the historical perspectives of different variants of OCT development can be found in the literature.^18^^,^^63^

PR-OCT measurements are a precursor of the modern wide-field QPI techniques; however, the phase stability in the presence of motion and the phase noise due to non-common path geometry pose limits to the sensitivity of the technique to the extracted phase information.^6^^,^^64^ Stable, quantitative phase measurements have been reported in the literature using the FD-OCT technique utilizing a common path length geometry, in which the incident light reflected from the sample coverslip serves as the reference field for interference with the light backscattered from the sample.^65^^,^^66^ Different detection schemes including multi-frame, phase-shifting methods as applied to OCT are detailed in Ref. 64.

**Fig. 12:**
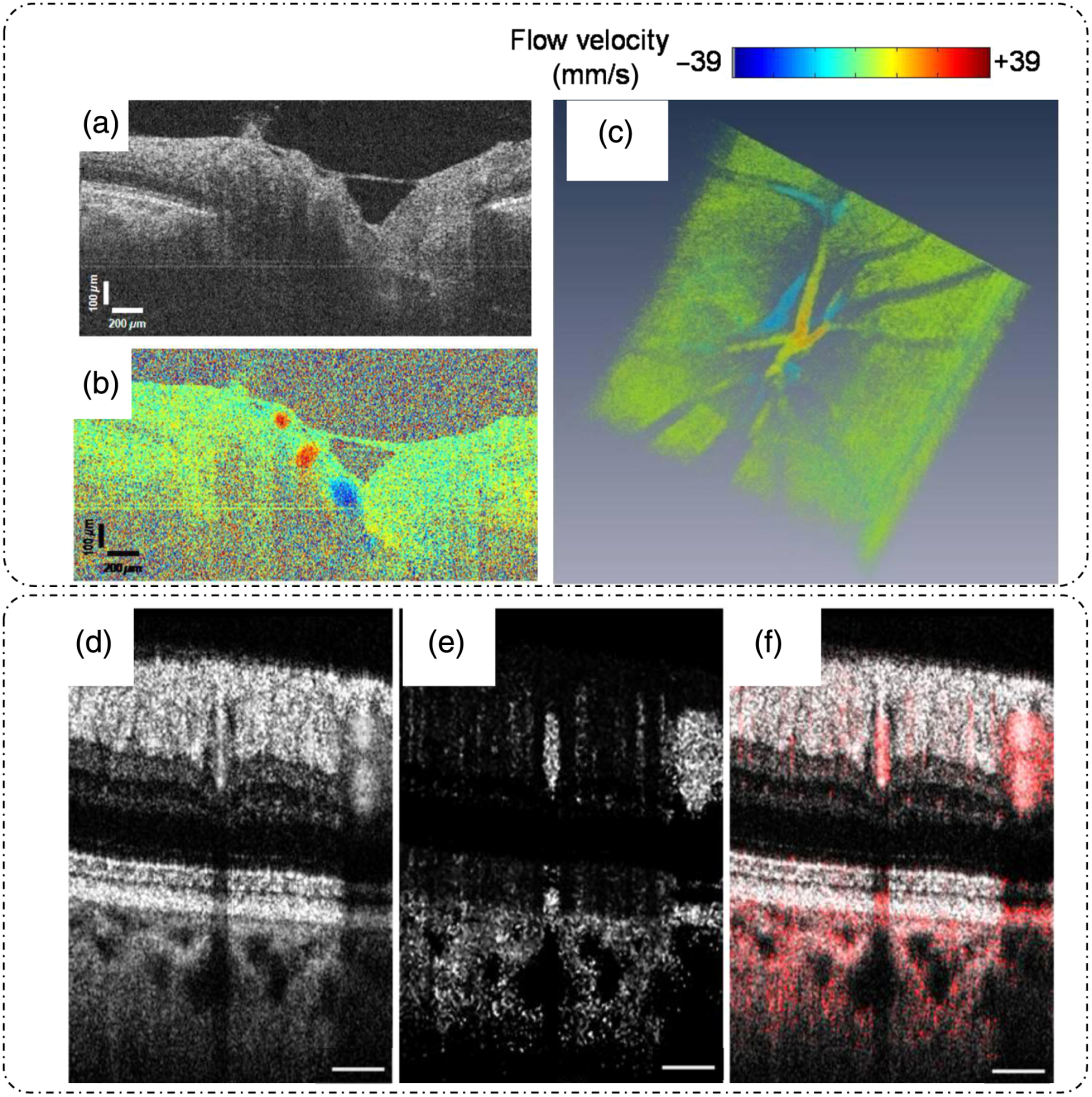
Applications of Doppler OCT and pvOCT. Doppler OCT: (a) retinal structural reflectivity B-scan, (b) Doppler OCT image at 200 kHz, and (c) 3D tomogram of Doppler OCT images at 200 kHz. Phase-variance OCT: (d) retinal OCT average intensity image, (e) phase-variance image, and (f) overlay map with average intensity in gray and phase variance in red. Scale bars for panels (d)–(f) are 250  μm. (a)–(c) Reproduced with permission from Ref. 20, © 2011, Optica; (d)–(f) reproduced with permission from Ref. 70, © 2011, Optica.

Phase-resolved optical Doppler tomography (PR-ODT) involves combining Doppler phenomena with OCT to enable quantitative flow imaging capabilities. The phase difference between two A-scans yields the Doppler frequency shift at a particular spatial location $\Delta \omega_{d}=\Delta \phi /T$, where T is the time duration between two A-scans.^67^^,^^68^ The axial flow-velocity is subsequently calculated as^69^ $v=\frac{\lambda_{0}\Delta \phi }{4\pi T}$. PR-ODT measurements are sensitive to the angle between the incidence (probe) beam and the direction of flow.^68^ An improvement over PR-ODT in terms of lower sensitivity to the incident angle was introduced in a technique that utilizes the variance in the Doppler frequency spectrum as a contrast mechanism to generate flow maps.^68^ Such techniques have been applied successfully to study the blood flow in the retina.^20^ Figure 12 shows one such example of retinal blood flow measurement using Doppler OCT with a B-scan image [Fig. 12(a)], a Doppler OCT image at 200 kHz [Fig. 12(b)], and a 3D tomogram of Doppler OCT images [Fig. 12(c)].^20^

The major limitation of Doppler OCT is that only the velocity component parallel to the illumination beam can be determined. Phase variance OCT (pvOCT) is a motion contrast technique that improves on this limitation.^71^ pvOCT uses B-scans (instead of A-scans in Doppler OCT) to determine the phase shift as^70^ $\Delta \phi_{i}(x,z,t)=\phi_{i+1}(x,z,t+T)-\phi_{i}(x,z,t)$. Phase variance is then calculated as^70^ $\sigma_{v}^{2}=\frac{1}{N-1}\overset{N-1}{\underset{i=1}{\sum }}[\Delta \phi_{i}(x,z,t)-\frac{1}{N-1}\overset{N-1}{\underset{i=1}{\sum }}\Delta \phi_{i}(x,z,t){]}^{2}$, where N is the number of B-scans. It is important to note that this technique is not quantitative in flow measurements and is a contrast enhancement technique for visualizing the motion of scatterers. Figures 12(d)–12(f) display the results of retinal imaging using pvOCT, showing average intensity image [Fig. 12(d)], phase variance image [Fig. 12(e)], and overlayed intensity (gray) and phase variance image (red) highlighting the blood vessels [Fig. 12(f)].^70^

**Fig. 13:**
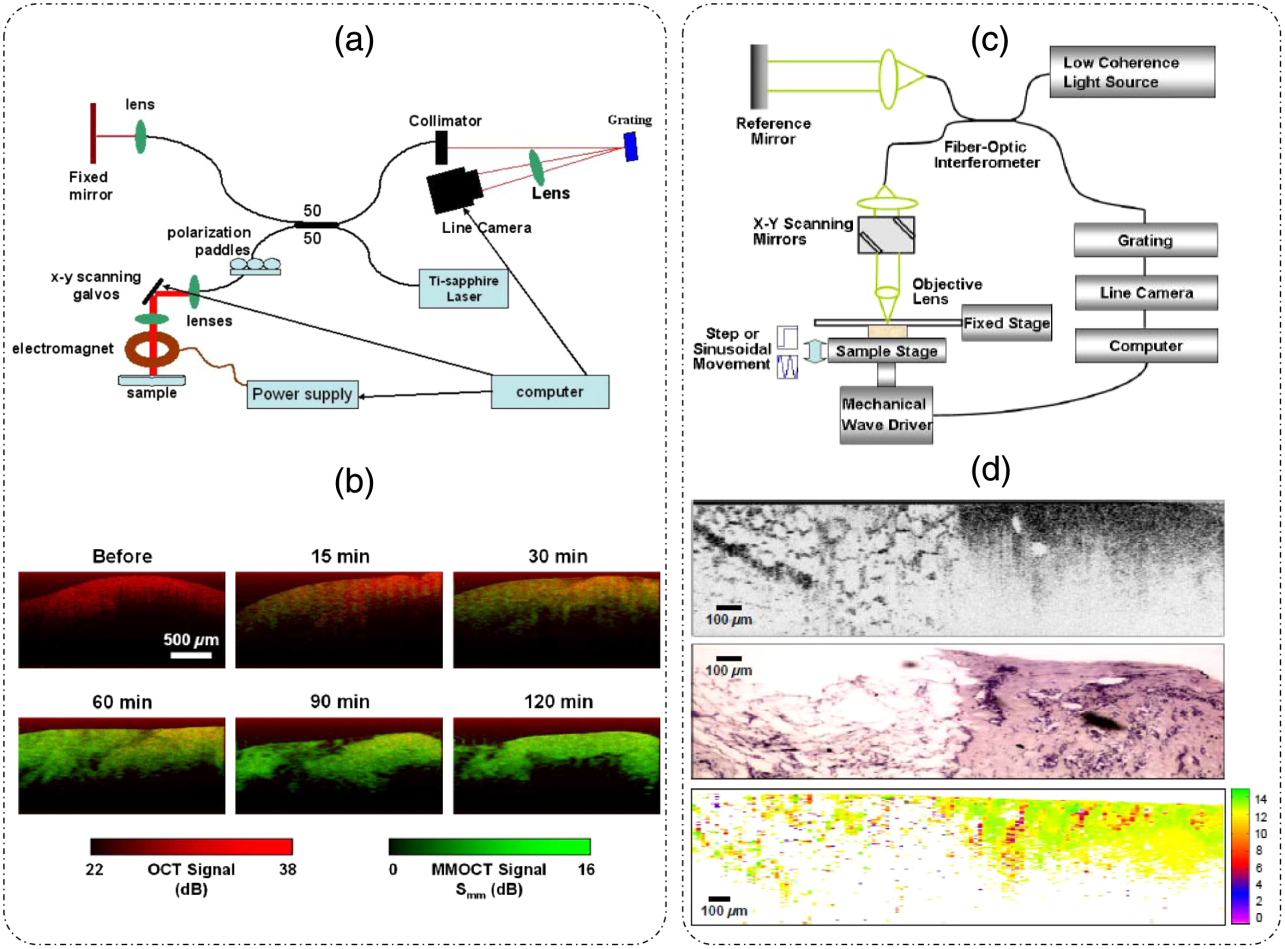
Dynamic OCT. (a) MMOCT setup—a standard SD-OCT system with electromagnet above the sample for magnetic field perturbations and (b) MMOCT application-timelapse images showing structural OCT (red) overlayed with MMOCT (green) signal in a rat tumor tissue sample, showing the diffusion of magnetic nanoparticles over time. (c) Setup for phase-resolved OCE—a standard SD-OCT system with excitations provided by the mechanical wave driver. (d) OCE application in breast tissue sample with normal adipose tissue on the left and tumor tissue on the right in each of the three images with the top image showing the B-mode structural OCT image, the middle row showing the corresponding histology image, and the bottom row showing the elasticity map (color bar in kilopascal). (a) Reproduced with permission from Ref. 72, © 2007, SPIE; (b) reproduced with permission from Ref. 19, © 2008, Optica; (c) and (d) reproduced with permission from Ref. 73, © 2008, Optica.

Magnetomotive OCT (MM-OCT) is another dynamic OCT technique that is used to study the dynamics of magnetic nanoparticles (a contrast agent) embedded in the biological sample.^19^^,^^72^ The dynamics is characterized by changes in both the amplitude and phase information obtained from PR-OCT techniques such as SD-OCT.^19^^,^^72^ Phase measurements are reported to provide highly sensitive dynamics information (∼8  nm sensitivity).^72^ The representative optical setup for MM-OCT is shown in Fig. 13(a), which is an SD-OCT system with an added computer-controlled electromagnet above the sample.^72^ The electromagnet is used to modulate the behavior of magnetic nanoparticles inside the sample. The motion of magnetic nanoparticles inside the sample can provide information about the mechanical properties of the sample, such as elastic modulus, viscosity, and Young’s modulus.^72^ Following Eq. (31), the B-scan measurement is expressed as^19^

$$\Gamma (x,z)=A(x,z)e^{i\phi (x,z)},$$ (32)

where z=ωτ/k with k is the wave number in the sample.^19^ Oldenburg et al.^19^ performed a B-scan with a simultaneous temporal modulation of the magnetic field, which modifies Eq. (32) to

$$\Gamma (x,z;t)=A(x,z;t)e^{i\phi (x,z;t)}\approx A(x,z)e^{i\phi (x,z)}e^{i2k\Delta z(x,z;t)}\;\forall \;\Delta z\ll l_{c}.$$ (33)

The resultant magnetomotive OCT (MMOCT) signal after background rejection was then expressed as^19^

Γmm(x,z)=10 log(f^on(x,z)|BPF{Don(x,z;t)}|f^off(x,z)|BPF{Doff(x,z;t)}|), (34)

where $D(x,z;t)=\frac{\partial }{\partial t}(\arg[\Gamma (x,z;t)])$, $\hat{f}(r)=(\frac{\cos(\phi (r))+1}{2})$ represents a phase filter. Here, BPF represents a band pass filtering operation about the magnetic field modulation frequency, and the subscripts on and off denote the B-mode image measurement with and without magnetic field modulation, respectively.^19^ Time-lapse images (OCT image in red overlayed with the MMOCT signal in green) of diffusion of magnetic nanoparticles within a rat tumor are shown in Fig. 13(b).^19^ Phase-resolved dynamic optical coherence elastography (OCE)^73^ is another technique that is similar in principle to MMOCT but involves external mechanical excitations applied to the sample. Such perturbations allow for the mechanical characterization (viscoelastic properties) of the sample under study. An example setup using an SD-OCT system with mechanical perturbations is shown in Fig. 13(c).^73^ The mechanical wave driver was operated in either step or sinusoidal mode and was used to excite the sample. Figure 13(d)^73^ shows the results of OCE performed on breast tissue samples containing tumor and normal adipose regions. The top image in Fig. 13(d) is the structural OCT B-mode image with normal tissue on the left and tumor tissue on the right; the corresponding histology image is shown in the middle row.^73^ The bottom row represents the elasticity map obtained from phase-resolved OCE with sinusoidal mechanical perturbation.^73^ The distinction between normal and tumor regions is clearly visible in the elasticity map, making OCE a promising method for diagnostics.^73^

### 2.3 Harmonic Optical Tomography

**Fig. 14:**
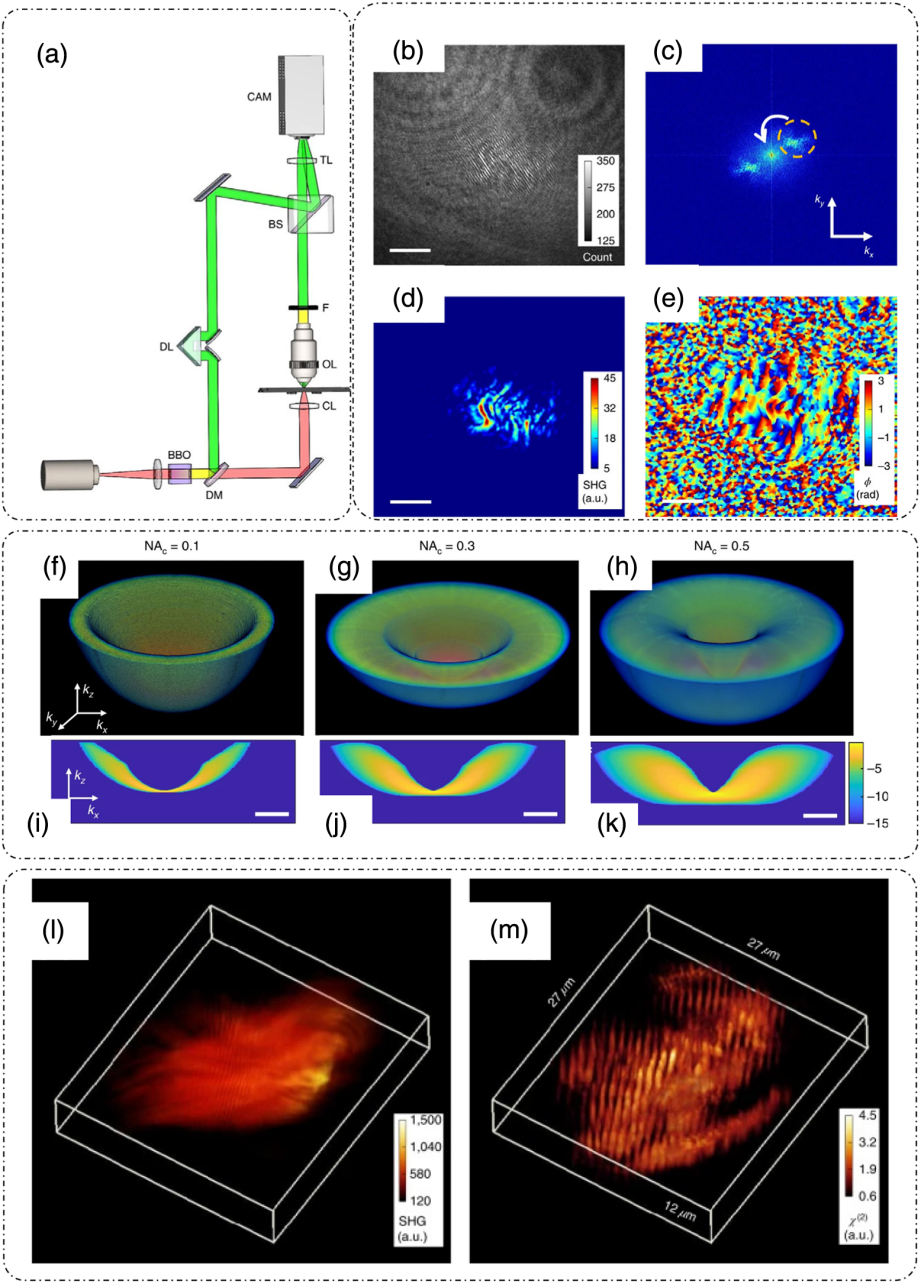
HOT. (a) Setup; BBO, beta-barium borate crystal; DM, dichroic mirror; DL, delay line; CL, condenser lens; OL, objective lens; TL, tube lens; BS, beam splitter; F, filter. (b) Measured hologram of a rabbit lymph node tissue slice. (c) Spatial frequency spectrum upon Fourier transform of panel (b). (d) Extracted amplitude and (e) phase of the SHG field. (f)–(h) System transfer function under varying illumination NA, (i)–(k) kx-kz views of the system transfer function in panels (f)–(h), respectively. The color bar is in the logarithmic scale with arbitrary units. Tomograms of the same field of view of murine muscle sample measured through (l) SHGM and (m) HOT. Scale bars for panels (b) and (d) are 10  μm, and those for panels (f)–(k) are $10\;\mathrm{rad}\;\mu m^{-1}$. (a)–(m) Reproduced with permission from Ref. 21, © 2020, The Authors, under exclusive license to Springer Nature Limited.

HOT is a method for estimating the second-order non-linear susceptibility distribution from an off-axis holographic microscopy setup based on SHGM.^21^ The experimental setup for HOT is shown in Fig. 14(a).^21^ Light from an assembly of a mode-locked Yb-fiber laser and a parabolic amplifier after passing through a non-linear crystal is divided into a fundamental and a harmonic component. The fundamental component of the incidence beam passes through the sample, where it is again split into a fundamental and a harmonic component due to the non-centrosymmetric nature of the sample. After the objective, a filter blocks the fundamental component and allows only the SHG field to pass through. This sample beam then interferes with the crystal-generated reference harmonic beam at the camera plane in an off-axis configuration, meaning that the reference and the sample beam have an angular offset between them.^21^ There are a few special considerations involved in this setup, which allows for widefield illumination and excellent depth sectioning. To achieve widefield illumination, the fundamental beam is defocused.^21^ The objective lens used is 100×/0.9NA, which increases the sectioning in conjunction with the wide-field illumination.^21^ To match the time of arrival of reference and sample beams at the camera plane, an optical delay is introduced in the light path of the harmonic reference beam.^21^

The acquired hologram, as shown in Fig. 14(b),^21^ is then processed. The reconstruction is based on recovering one of the sideband peaks in the Fourier spectrum of the hologram [Fig. 14(c)], as discussed earlier in part I of this review^8^ to extract the amplitude [Fig. 14(d)] and phase [Fig. 14(e)] of the complex SHG field.^21^ The sample is axially scanned to enable tomographic image formation.^21^ Below, we discuss the mathematical description of HOT imaging, following Ref. 21.

Consider the non-linear wave equation for an inhomogeneous medium given as^21^

$$\nabla^{2}U(r,t)-\frac{n^{2}(r)}{c^{2}}\frac{\partial^{2}U(r,t)}{\partial t^{2}}=\mu_{0}\frac{\partial^{2}P_{NL}(r,t)}{\partial t^{2}},$$ (35)

where U is the spatiotemporal complex field, n is the inhomogeneous RI, and $P_{NL}$ is the induced polarization that, for a non-linear interaction, is expressed in terms of second-order susceptibility $\chi^{\left(2\right)}$ as $P_{NL}=\varepsilon_{0}\chi^{\left(2\right)}(r)U^{2}(r,t)$.^21^

Substituting for $P_{NL}$ and taking the temporal Fourier transform, Eq. (35) becomes^21^

$$\nabla^{2}U(r,\omega )+n^{2}(r,\omega )\frac{\omega^{2}}{c^{2}}U(r,\omega )=-\frac{\omega^{2}}{c^{2}}\chi^{\left(2\right)}(r)[U(r,\omega ){Ⓥ}_{\omega }U(r,\omega )],$$ (36)

where ${Ⓥ}_{\omega }$ indicates convolution in the temporal frequency domain.

Applying the undepleted-pump approximation^74^ and the first-order Born approximation, Eq. (36) is decomposed into three components (see the supplementary section of Ref. 21 for a full derivation). The first is a linear scattering equation ^21^

$$\nabla^{2}U^{\omega_{0}}(r)+n^{2}(r,\omega_{0})\beta_{1}^{2}U^{\omega_{0}}(r)=0,$$ (37)

and the other two describe the unscattered SHG field and scattered harmonic field, given respectively as^21^

$$\nabla^{2}U_{\mathrm{SHG}}^{2\omega_{0}}(r)+({\overset{¯}{n}}_{2}\beta_{2}{)}^{2}U_{\mathrm{SHG}}^{2\omega_{0}}(r)=-\beta_{2}^{2}\chi^{\left(2\right)}(r)[U_{i}^{\omega_{0}}(r){]}^{2},$$ (38)

$$\nabla^{2}U_{s}^{2\omega_{0}}(r)+({\overset{¯}{n}}_{2}\beta_{2}{)}^{2}U_{s}^{2\omega_{0}}(r)=-\beta_{2}^{2}\chi (r)U^{2\omega_{0}}(r),$$ (39)

where $U_{\mathrm{SHG}}^{2\omega_{0}}$ is the second harmonic field generated by the non-linear sample, ${\overset{¯}{n}}_{2}$ is the RI averaged spatially, and $\beta_{2}=\frac{2\omega_{0}}{c}$.

For weakly scattering samples, the solution of Eq. (39) is neglected, and the SHG field for monochromatic plane wave incidence is expressed in the angular spectrum domain as^21^

$$U_{\mathrm{SHG}}^{2\omega_{0}}(k_{⊥},z)=\frac{\beta_{2}^{2}}{2\gamma_{2}}e^{i\gamma_{2}z}A_{o}(k_{⊥})A_{i}({k^{\prime }}_{i⊥})A_{i}({k^{\prime \prime }}_{i⊥})\chi^{\left(2\right)}(k_{⊥}-{k^{\prime }}_{i⊥}-{k^{\prime \prime }}_{i⊥},\gamma_{2}-\gamma_{1}^{\prime }-\gamma_{1}^{\prime \prime }),$$ (40)

where, $\gamma_{p}=\sqrt{({\overset{¯}{n}}_{p}\beta_{p}{)}^{2}-|k_{⊥}{|}^{2}}.$ for p=(1,2), $A_{i}$ is the 2D condenser aperture and $A_{o}$ is the objective pupil function.^21^

At the camera plane, the reference and SHG fields interfere with each other, and the resultant cross-spectral density is expressed as^21^

$$W(k_{⊥},k_{z})\approx \frac{\beta_{2}^{2}}{2\gamma_{2}}A_{0}(k_{⊥}+{k^{\prime }}_{i⊥}+{k^{\prime \prime }}_{i⊥})\delta [k_{z}-(\gamma_{2}-\gamma_{1}^{\prime }-\gamma_{1}^{\prime \prime })]A_{i}({k^{\prime }}_{i⊥})A_{i}({k^{\prime \prime }}_{i⊥})\chi^{\left(2\right)}(k_{⊥},k_{z}).$$ (41)

With consideration of all possible illumination angles, the instrument transfer function is expressed as^21^

$$H(k_{⊥},k_{z})=\int \beta_{2}^{2}W\left(\omega_{0}\right)P(k_{⊥},k_{z})⊗[U_{i}(k_{⊥},k_{z})ⓋU_{i}(k_{⊥},k_{z})]d\omega_{0},$$ (42)

where the 3D pupil function is specified as $P(k_{⊥},k_{z})=\frac{1}{2\gamma_{2}}A_{0}(k_{⊥})\delta (k_{z}-\gamma_{2})$. The dependence of H on the illumination condenser NA is shown in Figs. 14(f)–14(k).^21^ As the NA increases, the frequency coverage in the z direction increases, and hence, sectioning and the axial-resolution increase.^21^ The improvement in sectioning and the resolution that results from applying the HOT principle to SHGM can be seen in the tomographic rendering of murine muscle tissue measured through normal SHGM [Fig. 14(l)] and by applying HOT [Fig. 14(m)].^21^ The improvement is drastic as the striated appearance of the muscle tissue is clearly seen in the HOT reconstruction.^21^ The study of non-centrosymmetric structures that normally benefit from the SHGM setup can utilize the HOT principle to obtain much better tomographic information due to the improvement in sectioning capability. These samples include collagens that are of significant importance in cancer research, as mentioned earlier.

### 2.4 Interferometric Synthetic Aperture Microscopy

ISAM is a technique in which the scattered light from the object is utilized for solving the inverse problem.^22^ The solution that incorporates the scattering and diffraction effects while considering a Gaussian beam profile allows for the determination of the three-dimensional structure of the object. This technique provides spatially invariant resolution at any depth of focus inside the object.^22^

**Fig. 15:**
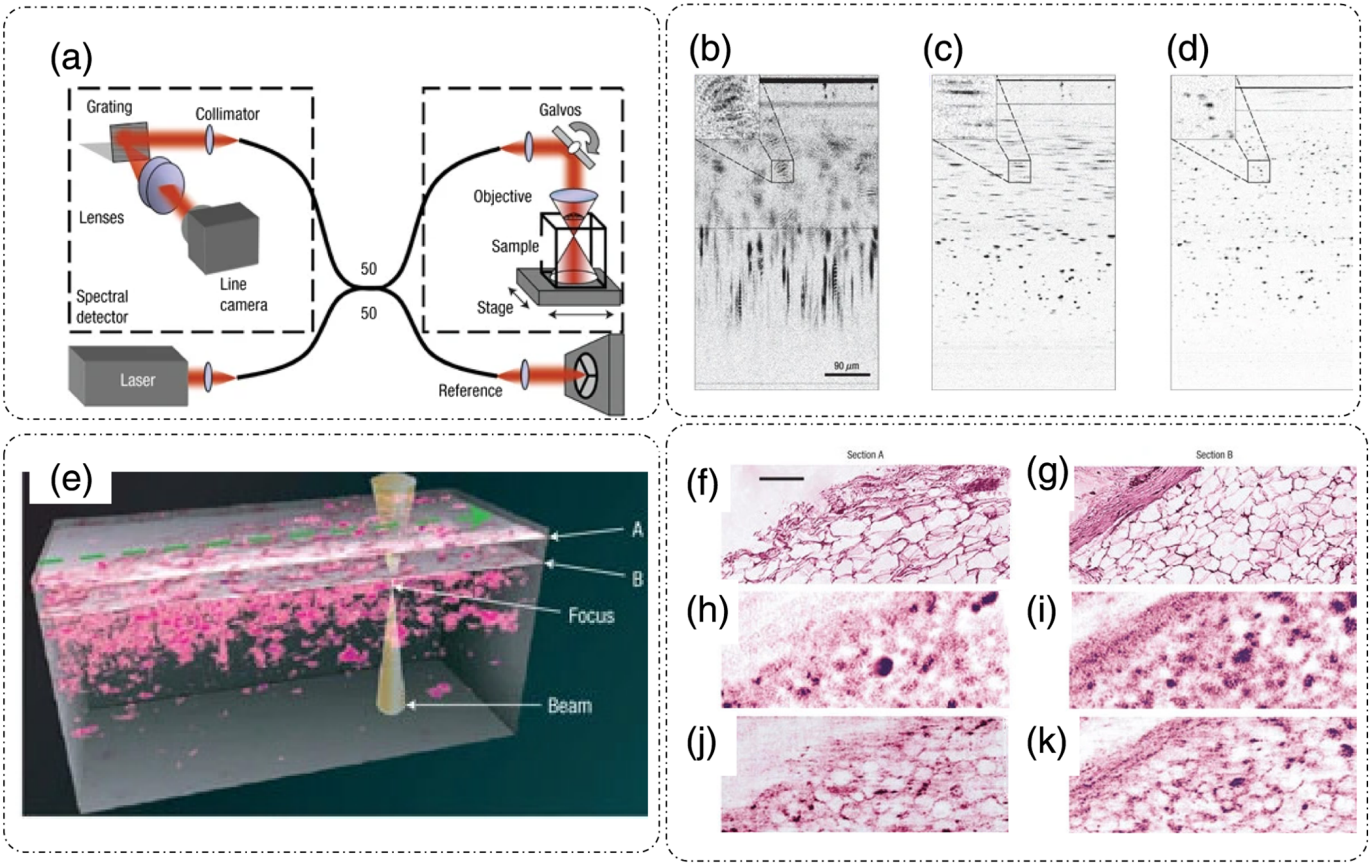
Interferometric scanning aperture microscopy (ISAM). (a) Setup, (b) raw cross-section of a tissue phantom, (c) dispersion compensated image, and (d) final ISAM reconstructed image. (e) Representation of sample planes A and B selected for ISAM imaging. (f), (g) Histological images for the two sample planes for comparison. (h), (i) Raw interferometric images (j), (k) ISAM reconstructed images. Scale bars for panels (b)–(d) are 90  μm and for panels (f)–(k) are 100  μm. (a)–(k) Reproduced with permission from Ref. 22, © 2007, Springer Nature Limited.

The general setup for ISAM is shown in Fig. 15(a),^22^ where light from a coherent femtosecond pulsed source is fed into a Michelson interferometer. The illumination beam is split by a coupler into reference and sample arms. The reference beam is reflected toward the output port of the coupler into the line camera. The sample beam travels through the objective to the sample, is scattered and collected by the objective, and is sent to the output port where it interferes with the reference beam. The sample can be scanned either through a stage assembly or the illumination beam can be scanned through a Galvano-mirror assembly.^22^ The spectral interferometric assembly at the output port is used to detect the cross-correlation between the reference and sample beams through the captured cross-spectral density at the line camera. This measurement of cross-spectral density and hence the cross-correlation results in the determination of a complex scattered field.^22^

The mathematical description follows a similar formalism as discussed at the beginning of Sec. 2. However, the beam is assumed to possess a Gaussian profile. The signal detected at the camera plane is expressed in k-domain as^22^

$$\overset{˜}{S}(k_{⊥},\beta )=A(\beta )[\frac{i2\pi^{2}}{k_{z}(k_{⊥}/2)}\frac{\beta^{2}}{\alpha^{2}}e^{-\frac{\alpha^{2}k_{⊥}^{2}}{4\beta^{2}}}]\overset{˜}{\chi }(k_{⊥},-2k_{z}(k_{⊥}/2)),$$ (43)

where the source power spectral density is $A^{2}(\beta )$, $\alpha =\pi /{\mathrm{NA}}_{\text{objective}}$, the exponential term is the 2D Fourier transform of the input beam profile $g(r,\beta )\propto e^{-\frac{r^{2}}{2W^{2}(\beta )}}$ and W(β)=α/β.^22^ The rest of the symbols carry the same meaning as discussed previously in this paper.

Equation (43) relates the measured 2D cross-spectral density signal to the frequency components of the 3D Fourier transform of the object’s susceptibility. Interpolation of measured 2D slices using the Fourier slice theorem and back projection algorithm is used using an elliptical resampling grid to recover the maximum allowed 3D frequency space of the object.^22^ There were some special steps implemented in both the reconstruction algorithm and the hardware to improve the performance. On the algorithm side, a dispersion compensation method was employed.^22^ On the hardware end, because this is not a common path geometry, a reference derived from the sample itself (reflection from the top coverslip on the sample) was used to correct for phase instability.^22^

Hence, ISAM can provide high spatially invariant resolution at significant depths, reaching ∼2  mm inside the sample, as shown in Figs. 15(b)–15(d).^22^ Figure 15(b) shows a raw cross-section profile of a tissue phantom.^22^ After dispersion compensation, the result is shown in Fig. 15(c).^22^ Figure 15(d) shows the final ISAM reconstruction result in which individual scatterers can be resolved throughout the depth of the sample.^22^ Figures 15(e)–15(k) illustrate the ISAM imaging results for a human breast tissue.^22^ Two sections marked A and B, shown in Fig. 15(e), were selected for imaging.^22^ Figures 15(h) and 15(i) show *en face* planes of the raw unprocessed data, which transforms to Figs. 15(j) and 15(k) after ISAM processing.^22^ These results correspond well with the histological images shown in Figs. 15(f) and 15(g).^22^

Because ISAM can provide clearly resolved scatterers irrespective of the axial position inside the sample, it presents great opportunities for tissue imaging *in vivo*.^22^

### 2.5 Computational Adaptive Optics (CAO) and ISAM

Aberrations are a phenomenon associated with an optical system and sometimes the (highly scattering) sample itself; they result in image distortions, such as loss of high spatial-frequency information, asymmetric PSF, and SNR degradation. As discussed in DHM in part I of this review,^8^ many different techniques exist for aberration correction applied either in real time during acquisition or as a post-acquisition processing technique. Aberrations pose a greater risk for 3D imaging of highly scattering samples as the effects become more pronounced for deeper sections of such samples. In Sec. 2.4, we discussed that ISAM can provide spatially invariant resolution at any depth within the sample; however, effects of other aberrations, such as astigmatism, can still introduce significant distortions.^23^ Adie et al.^23^ addressed this problem using computational aberration correction (by introducing a technique called computational adaptive optics-CAO) in conjunction with ISAM. They demonstrated the SNR and resolution improvement in OCT and ISAM using a post-acquisition aberration correction algorithm in which the phase aberrations are modeled using Zernike polynomials and corrected by adjusting the phase-profile of a virtual pupil-plane. This pupil-plane phase profile is obtained by a transverse Fourier transform of the complex field (which in turn represents the complex PSF of the system when sub-diffraction samples are measured) at the focal plane.^23^

The 3D aberration correction filter based on the Zernike polynomials is expressed as^23^

$$H_{3D}(k_{⊥};k)=e^{-i\frac{k}{k_{c}}\;\arg\{H_{2D}(k_{⊥})\}},$$ (44)

where $H_{2D}(k_{⊥})=e^{-ik_{c}\phi_{h}(-2\pi z_{f}k_{x}/k_{c},-2\pi z_{f}k_{y}/k_{c})}$ is the 2D aberration correction filter at focal length $z_{f}$, $k_{c}$ is the central wavenumber, and $\phi_{h}$ is the phase deviation of focal-plane PSF from the uniform transverse-frequency phase.^23^

**Fig. 16:**
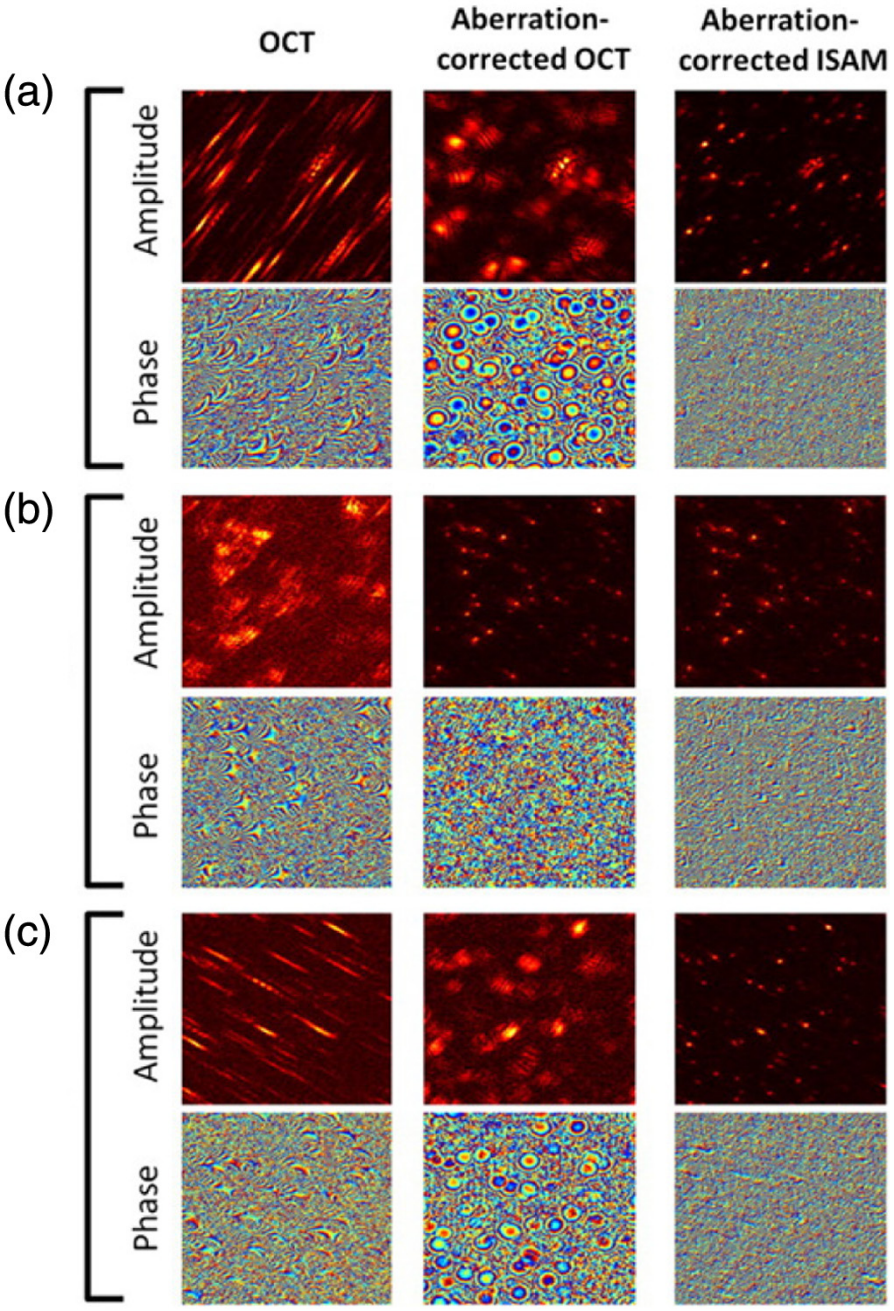
CAO for OCT and ISAM: the first column shows the raw and aberrated amplitude and phase information of a silicone phantom sample measured with OCT. The second column shows aberration-corrected results after the application of CAO, and the third column shows the CAO-ISAM results for (a) z=300  μm, (b) z=0  μm, and (c) z=−300  μm. Image dimensions are 256  μm×256  μm. (a)–(c) Reproduced with permission from Ref. 23, © 2012, PNAS.

Figure 16 shows the results of the CAO-ISAM processing applied on a silicone phantom dataset that comprises OCT images of sub-diffraction titanium dioxide (${\mathrm{TiO}}_{2}$) scatterers, imaged using an astigmatic beam.^23^ The effects of aberrations are evident in the first column of Fig. 16 on both amplitude and phase information at varying depths within the sample (z=300  μm [Fig. 16(a)], 0  μm [Fig. 16(b)], and −300  μm [Fig. 16(c)]).^23^ After the application of the CAO algorithm, the astigmatism is evidently removed, as observed in the middle column of Fig. 16.^23^ The scattering potential obtained after ISAM is shown in the last column of Fig. 16, which shows a spatially invariant resolution at all three depths.^23^

CAO-based ISAM enables aberration-corrected, spatially invariant resolution at all imaging depths within a highly scattering sample. This is a very promising approach for broadband, interferometric tomography as no additional optical elements such as wavefront sensors or digital micromirrors are required for aberration estimation and correction.^23^

### 2.6 Tomographic Phase Microscopy

Most of the 3D QPI techniques discussed in this review utilized scattering theory to formulate the inverse problem and determine the RI distribution of the object.

There is another class of methods for 3D QPI of thin samples, which is analogous to X-ray tomography, in which projections along straight lines are used to estimate the object structure. These techniques rely on the principle that, for the weakly scattering and thin samples, the optical phase delay measured through forward scattering is proportional to the projection of the RI along a straight line passing through the depth of the object. Using this approximation, one can use projections of the RI to estimate the 3D structural information of the object. Reference 24 proposed a 3D QPI technique called TPM based on this projection formalism of the RI for thin objects. Here, we discuss the principle of operation and reconstruction technique of TPM, as introduced in Ref. 24.

**Fig. 17:**
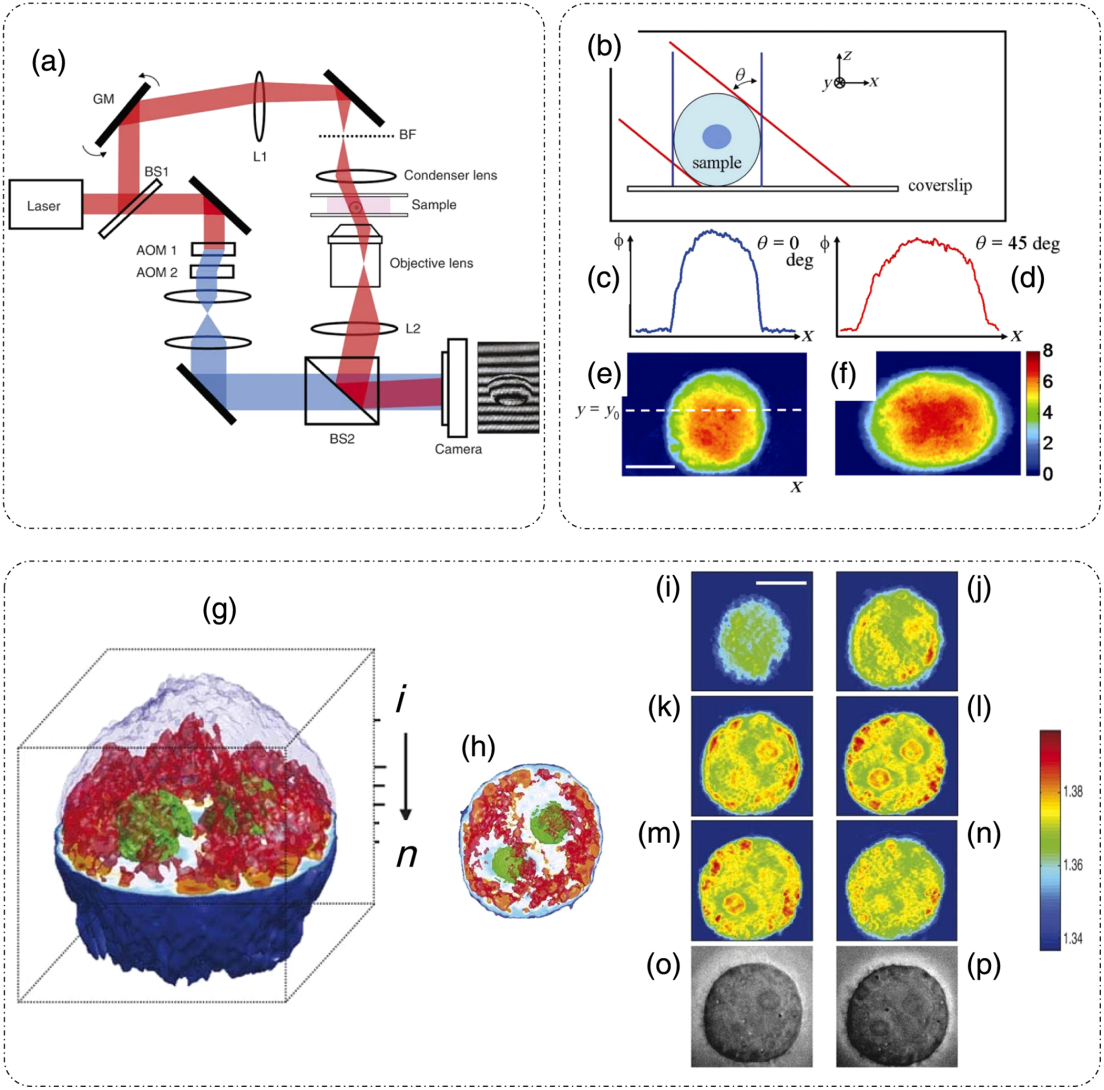
TPM. (a) Setup. BS, beam splitter; GM, galvano mirrors; AOM, acousto-optic modulators; L, lens; BF, condenser back focal plane. (b) Phase-projection geometry. (c), (d) Projection phase line profiles through the position of dotted lines in panels (e) and (f). (e), (f) Projection phase maps under varying angles of illumination. (g) RI tomograms of HeLa cell with a cube length of 20  μm; (h) top view of tomogram in panel (g); (i)–(n) sections of RI tomograms in panel (g). (o), (p) Brightfield images for comparison at the focus corresponding to (k)–(l) respectively. Scale bars for panels (e), (f), (i)–(p) indicate 10  μm. The color bar in panels (e) and (f) represents a phase in radians, and that in panels (i)–(n) represents an RI at 633 nm. (a)–(p) Reproduced with permission from Ref. 24, © 2007, Springer Nature America, Inc.

Figure 17(a) shows the heterodyne Mach–Zehnder type interferometric optical setup for TPM.^24^ An illumination beam from a HeNe laser (633 nm) is divided into a reference and a sample beam by a beam splitter. The reference arm contains two acousto-optic modulators that shift the frequency of the reference beam by 1250 Hz. This frequency-modulated reference beam is then spatially filtered and directed toward the camera plane through another beam splitter. The sample beam is tilted at different angles through a galvano mirror. The range of illumination angles covered depends on the NA of the imaging system. After passing through the sample, the sample beam is directed by the final beam splitter onto the camera plane where it interferes with the frequency-shifted reference beam.^24^

To extract the phase information through the above-mentioned setup, four phase-shifted images are captured at the camera plane for each angled illumination.^24^ This temporal phase shifting is achieved by measuring the successive frames at a delay of 200  μs, which produces a phase delay of π/2 between successive frames. Phase is extracted using a phase-shifting algorithm and corrected for phase wrapping.^24^ The filtered back-projection algorithm is used for tomographic reconstruction.^24^ Figure 17(b) illustrates the geometry of back-projection, and the corresponding phase line profiles at a specific y-coordinate and image slices of an HeLa cell under different angles of illumination are shown in Figs. 17(c)–17(f).^24^ A tomographic reconstruction of an HeLa cell along with slices at different depths and a comparison with brightfield images are shown in Figs. 17(g)–17(p).^24^

The validity of the back-projection algorithm becomes questionable for highly scattering samples, specifically with depths greater than ∼15  μm.^24^ To solve this issue, Ref. 24 presented a method to axially scan the objective and capture the in-focus tomograms at different depths within the object. For the case of an ensemble of polystyrene beads in an optically cured adhesive, sandwiched between two coverslips, Choi et al.^24^ observed that, away from the plane of focus, the tomograms show diffraction artifacts, which can be removed by moving the objective to the focus of that part of the sample and acquiring the tomogram at the specific focus plane.^24^

## 3 Dynamics

### 3.1 Dynamic Light Scattering

DLS or photon correlation spectroscopy involves the study of properties of systems that have moving scatterers.^25^^,^^28^ Examples of such systems relevant to biology can be a solution of nanoparticles, proteins, etc.^26^^,^^27^ Here, we briefly discuss the mathematical formulation for DLS, and more detailed theoretical formulations can be found in the literature.^25^^–^^28^^,^^75^

Such a system can be mathematically modeled as an ensemble of particles, as discussed for static/elastic light scattering in part I of this review^8^ but with time-dependent positions. The dynamic scattering potential for a system of “N” identical but dynamic scatterers is then expressed as^6^

$$F(r,t)=F_{0}(r){Ⓥ}_{r}\underset{j}{\sum }\delta [r-r_{j}(t)],$$ (45)

where $F_{0}(r)$ is the scattering potential of a single scatterer and $r_{j}(t)$ is the time-dependent position of the j’th particle.

The scattering amplitude is then defined as^6^

$$\overset{˜}{F}(q,t)={\overset{˜}{F}}_{0}(q).\underset{j}{\sum }e^{iq\cdot r_{j}(t)},$$ (46)

where q is the scattering wavevector defined previously. The temporal field autocorrelation of dynamic scattered fields for a specific q is expressed as

$$\Gamma (q,\tau )={\left\langle \overset{˜}{F}(q,t)\cdot {\overset{˜}{F}}^{*}(q,t+\tau )\right\rangle }_{t}=|{\overset{˜}{F}}_{0}(q){|}^{2}{\left\langle \underset{m,n}{\sum }e^{iq\cdot [r_{n}(t)-r_{m}(t+\tau )]}\right\rangle }_{t}.$$ (47)

Because the random movement of sparse particles is mostly uncorrelated, the temporal average in Eq. (47) exists only for the same particle at different times, i.e., for m=n. Also, substituting $\left|{\overset{˜}{F}}_{0}(q){|}^{2}=\sigma_{d}(q\right)$ as the scattering differential cross-section, Eq. (47) is simplified to^28^

$$\Gamma (q,\tau )=N\sigma_{d}(q){\left\langle e^{iq\cdot [r(t)-r(t+\tau )]}\right\rangle }_{t}.$$ (48)

The normalized temporal field autocorrelation function is then given by

$$g_{1}(q,\tau )={\left\langle e^{iq\cdot [r(t)-r(t+\tau )]}\right\rangle }_{t}.$$ (49)

The field autocorrelation is related to the intensity autocorrelation by the Siegert relationship as^76^

$$g_{2}(\tau )=1+\alpha [g_{1}(\tau ){]}^{2},$$ (50)

where α is the coherence factor.

Considering the system of particles to be under Brownian motion, the probability distribution function of the displacements follows a homogenous diffusion equation, given as^28^

$$D\nabla^{2}\zeta (r,t)-\frac{\partial }{\partial t}\zeta (r,t)=0,$$ (51)

where D is the translational diffusion coefficient and ζ is the probability distribution function of particle translations.

Expressing Eq. (51) in the spatial frequency domain and solving for $\tilde{\zeta }$ gives

$$\overset{˜}{\zeta }(q,t)=e^{-Dq^{2}t}.$$ (52)

Using this result in Eq. (49) provides the first-order temporal field autocorrelation for diffusive particles:^26^

$$g_{1}(q,\tau )=e^{-Dq^{2}\tau }=e^{-\tau /\tau_{0}},$$ (53)

where $\tau_{0}=\frac{1}{Dq^{2}}$ is the decay time of the field autocorrelation.^28^

Hence, the measured temporal intensity autocorrelation is expressed as

$$g_{2}(q,\tau )=1+e^{-2Dq^{2}\tau },$$ (54)

and the diffusion coefficient is extracted by the curve fitting of the measured data using Eq. (54).

From the estimate of D, the particle hydrodynamic radius is estimated using the Stokes–Einstein relation as^26^

$$D=\frac{k_{B}T}{6\pi \eta a}.$$ (55)

Here, $k_{B}$ is the Boltzmann’s constant, T is the absolute temperature, η is the viscosity of the surrounding media, and a is the radius of the particle.

Substituting Eq. (55) in the definition of field decay time gives $\tau_{0}=\frac{6\pi \eta a}{q^{2}k_{B}T}$.

**Fig. 18:**
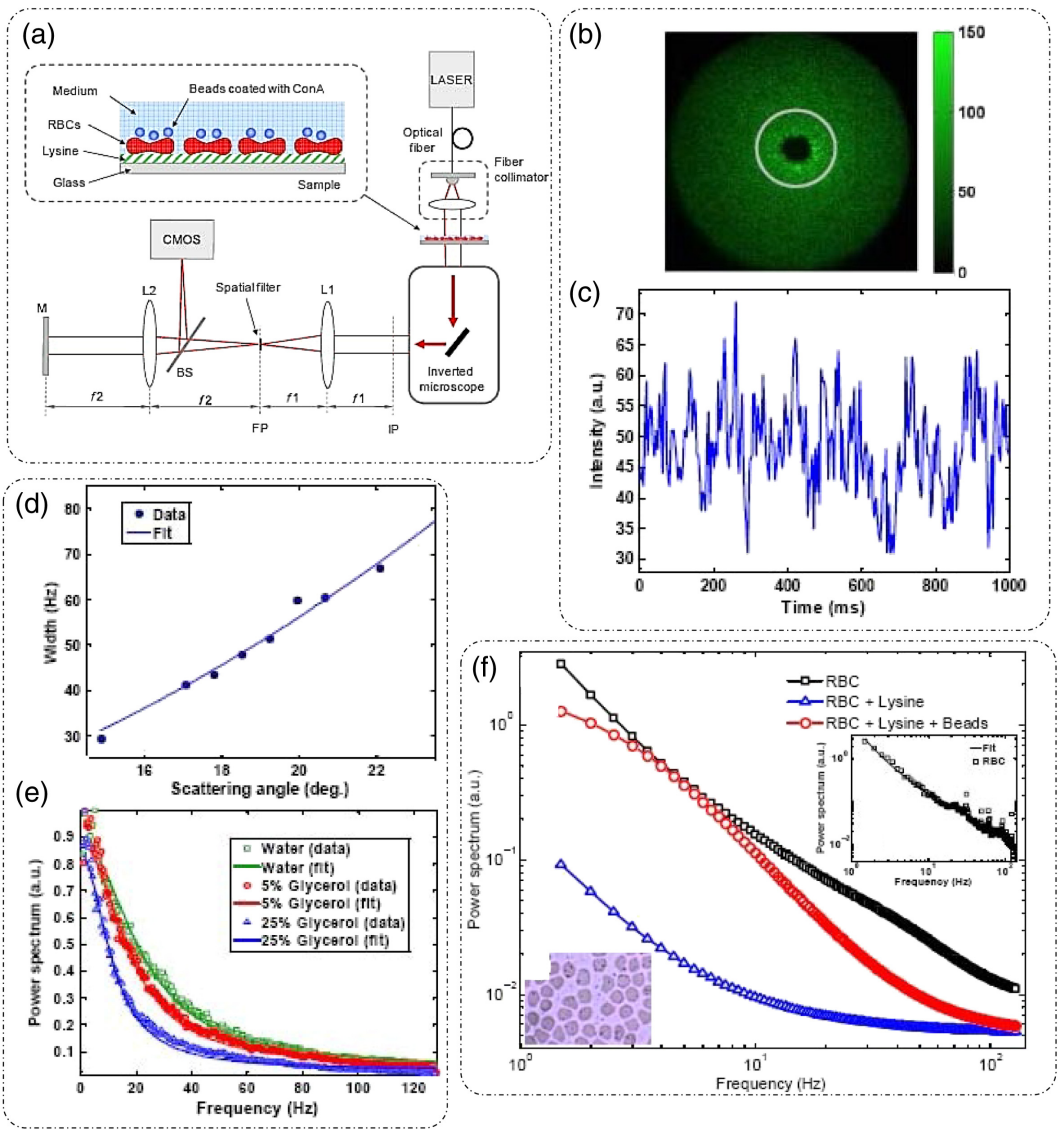
DLS and DSM. (a) optical setup for DSM. L, lens; M, mirror; FP, Fourier plane; IP, image plane; BS, beam splitter; CMOS, complementary metal-oxide semiconductor. (b) High-pass filtered scattered field/angular scattering map. (c) Temporal fluctuations of scattered intensity on a point on the white ring in panel (b). (d) Width of the power spectrum versus scattering angle. (e) Power spectrum of a system of microbeads in different solvents. (f) Power spectrum differences between adhered and unadhered RBCs. (a)–(f) Reproduced with permission from Ref. 77, © 2007, Optica.

Figure 18(a) shows the experimental setup for dynamic scattering microscopy (DSM) that utilizes DLS to study dynamics of membrane fluctuations in RBCs.^77^ In the DSM setup, an illumination beam from a laser is collimated through a fiber collimator and is fed into a standard inverted microscope. At the image plane, lens L1 produces a Fourier transform of the image field at plane FP where a spatial filter is used to block the DC. The resultant high-passed or DC removed scattered field at plane FP is imaged onto the CMOS camera by a folded 4f geometry implemented by lens L2 and mirror M.^77^ DLS measurements from the reported DSM setup were carried out on a sample of polystyrene beads immersed in glycerol solution of varying concentration.^77^ The results are shown in Fig. 18(b), which is the scattered field for one time frame with the DC part removed.^77^ The scattered intensity temporal fluctuations for a point on the ring in Fig. 18(b) are shown in Fig. 18(c).^77^ As discussed for FTLS in part I of this review,^8^ the angular scattering is obtained from the power spectra by averaging over rings of constant scattering wavevector q. Consider a Lorentzian distribution of the power spectra that is expressed as^77^

$$P(\omega )=\frac{A}{\Delta \omega }\frac{1}{1+(\omega /\Delta \omega {)}^{2}},$$ (56)

where A is the amplitude and $\Delta \omega =Dq^{2}$ is the width of the distribution. The scattering wavevector and angle of scattering are related as $q=2\beta_{0}\;\sin(\frac{\theta }{2})$. A plot of the width of the power spectrum versus scattering angle is shown in Fig. 18(d).^77^ Power spectra for microbeads (radius = 130 nm) in different solvents are shown in Fig. 18(e), where the viscosity of the solvent can be extracted from the width of the power spectra.^77^

To demonstrate the potential of DSM for characterizing DLS in biological samples in Ref. 77, protein Concanavalin A-coated polystyrene beads were attached to the membrane of RBCs adhered to the substrate using polylysine. Adhering RBCs to the substrate with polylysine ensured that the scattering signal is due to the dynamics of beads adhered to the membrane rather than whole cell translation, as shown by the power spectrum width of adhered and unattached RBCs in Fig. 18(f).^77^

Using the Siegert relationship in Eq. (50), one can estimate the temporal field autocorrelation function $g_{1}(\tau )$ from the measured temporal intensity autocorrelation function $g_{2}(\tau )$, which in turn is calculated from the power spectrum P(ω). The mean squared displacement (MSD) $\left\langle \Delta r^{2}(\tau )\right\rangle$ is evaluated from $g_{1}(\tau )$ using the following relation [from Eq. (49)]:^28^^,^^77^

$$\langle \Delta r^{2}(\tau )\rangle =-\frac{6}{q^{2}}\;\ln[g_{1}(\tau )].$$ (57)

Using the fluctuation-dissipation theorem and generalized Stokes–Einstein relation, the shear modulus was expressed in Ref. 77 as

$$G(\omega )=\frac{1}{6\pi aL(\omega )},$$ (58)

where a is the radius of the scatterers, Im[L(ω)]=ω2kBT⟨Δr2(ω)⟩, and the real part of L(ω) is calculated using Kramers–Kronig relation.^77^ It was shown in Ref. 77 that the membrane acts as a viscoelastic fluid based on the dependence of $G^{\prime \prime }\propto \omega^{0.69}$, obtained upon fitting the experimental data.

### 3.2 Diffusing-Wave Spectroscopy

DLS is modeled for sparse and thus weakly scattering systems.^26^^,^^28^^,^^75^ To account for light scattering through a densely populated mixture of scatterers, or in other words through a multiple scattering medium, the DWS technique can be applied to determine the dynamics of the scatterers on a length scale much smaller than the wavelength of light in the medium.^29^^,^^30^ DWS applies to light propagation through strong scattering solutions under the diffusion approximation.^29^

The temporal field autocorrelation function now assumes multiple scattering events and is expressed as^29^

$$g_{1}(\tau )=\overset{\infty }{\underset{n=1}{\sum }}P(n)e^{-\frac{2\tau }{\tau_{0m}}(\frac{l}{l_{t}})n},$$ (59)

where the summation is over all possible scattering paths of different orders. The quantity $\tau_{0m}=\frac{1}{D\beta_{0}^{2}}$ is the decay rate for multiple scattering that involves averaging over q for the decay rate $\tau_{0}$ associated with single scattering events in DLS, $l_{t}$ is the transport mean free path, and P(n) is the fraction of total scattered intensity that is scattered in the n’th scattering path.^29^

Interested readers can refer to Ref. 29 for a detailed mathematical analysis of DWS techniques and geometries. A technique that relates to intermediate scattering effects between DLS and DWS is reported in Ref. 75.

### 3.3 Dispersion Relation Phase Spectroscopy (DPS)

Dispersion relation phase spectroscopy (DPS) is a computational technique that utilizes the phase information from quantitative phase images to characterize intracellular mass transport in biological specimens.^31^ Here, we discuss the mathematical formulation of DPS as reported in Ref. 31.

The goal of DPS is the estimation of the diffusion coefficient and mean advection velocity through QPI measurements. The dry mass density^6^ satisfies the advection-diffusion equation

$$D\nabla^{2}\rho (r,t)-v\cdot \nabla \rho (r,t)-\frac{\partial }{\partial t}\rho (r,t)=0,$$ (60)

where ρ is the dry mass density, v is the advection velocity, and D is the diffusion coefficient. We express the spatiotemporal autocorrelation function as^31^

$$g(r_{0},\tau )={\left\langle \rho (r,t)\rho (r+r_{0},t+\tau )\right\rangle }_{r_{0},t}.$$ (61)

Upon performing the spatial Fourier transform of Eq. (61) and averaging over all realizations of the advection velocity, the temporal autocorrelation is expressed as^31^

$$\overset{˜}{g}(q,\tau )={\left\langle e^{iq.v\tau -Dq^{2}\tau }\right\rangle }_{v}.$$ (62)

Wang et al.^31^ assumed a Lorentzian probability distribution function for the advection velocity, with a mean $v_{0}$ and width Δv. In this case, Eq. (62) is simplified to^31^

$$\overset{˜}{g}(q,\tau )=e^{iq.v_{0}\tau }e^{-Dq^{2}\tau -q\Delta v\tau }.$$ (63)

**Fig. 19:**
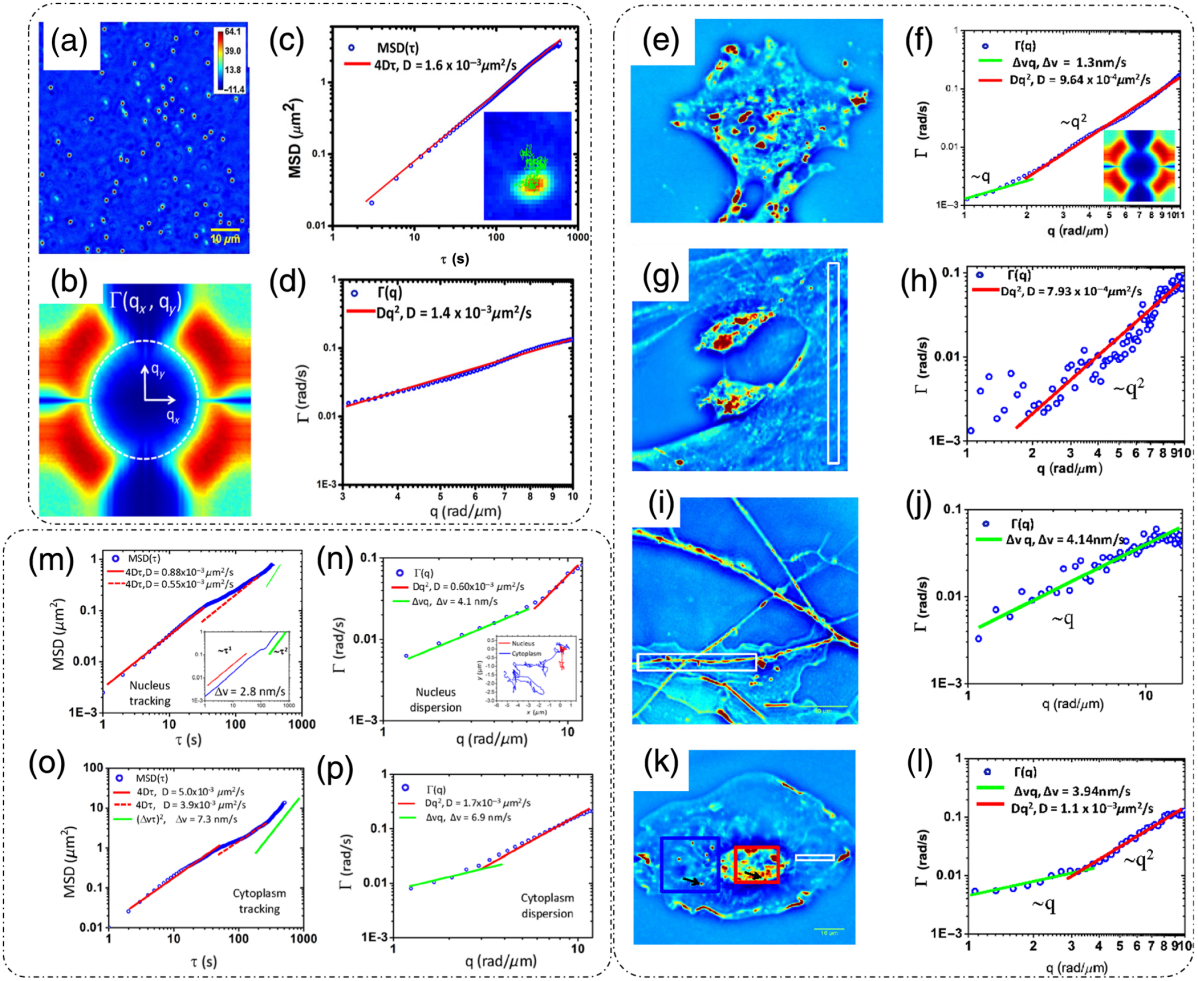
DPS. (a) SLIM image of 1  μm polystyrene beads, the color bar indicating path length in nanometers, (b) decay rate $\Gamma (q_{x},q_{y})$ versus spatial mode of panel (a); (c) MSD curve computed through particle tracking in panel (a), (d) DPS curve obtained by azimuthal averaging of panel (b) for beads sample. (e)–(k) SLIM images of various parts of cells marked by white boxes and different cell types, (f)–(l) corresponding DPS curves. (m), (o) Particle tracking results. (n), (p) DPS results for particles inside the blue (cytoplasm) and red (nucleus) boxes in panel (k). D, diffusion coefficient; Δν, spread of advection velocity; q, scattering wavevector. (a)–(p) Reproduced with permission from Ref. 31, © 2011, Optica.

From Eq. (63), it can be inferred that the decay rate of the temporal autocorrelation is $\Gamma (q)=Dq^{2}+\Delta vq$. In addition, the temporal autocorrelation is modulated by a sinusoid with a frequency $q\cdot v_{0}$ of the diffusion coefficient D, and the spread of advection velocity distribution Δv is extracted from the measured temporal autocorrelation data by fitting them with Eq. (30). Figure 19(a) shows the measured optical path length map of 1  μm polystyrene beads.^31^ The computed temporal decay rate is shown in Fig. 19(b).^31^ For validating DPS results, Wang et al.^31^ temporally tracked the beads, and the averaged MSD is shown in Fig. 19(c); linear curve fitting of this trend gave a diffusion coefficient value of $D=(1.60\pm 0.04)\times 10^{-3}\;\mu m^{2}/s$. Performing an azimuthal average of Fig. 19(b), Γ(q) was extracted and is shown in Fig. 19(d).^31^ A quadratic curve-fit was performed^31^ to estimate the diffusion coefficient value $D=(1.40\pm 0.07)\times 10^{-3}\;\mu m^{2}/s$. This value of the diffusion coefficient calculated using DPS closely matched with the particle tracking results. These results demonstrated the applicability and suitability of the DPS technique for measuring intracellular mass transport without explicit segmentation and subsequent tracking of the particles from the image.^31^ Figures 19(e)–19(l) show the dispersion curves for whole cell [Figs. 19(e) and 19(f)] and parts of cells denoted by white boxes in Figs. 19(g)–19(l).^31^ These dispersion curves show that, at low scattering frequencies (or high spatial ranges), the transport is mainly directed and deterministic, as shown by linear green curves, whereas for high scattering frequencies (low spatial ranges), the transport is diffusive or random, as shown by quadratic red curves.^31^

The transport of particles inside the boxed region in Fig. 19(k) in both the nucleus and cytoplasm was measured with both the particle tracking method and DPS.^31^ The results are shown in Figs. 19(m)–19(p),^31^ which shows that the diffusion coefficient and advection velocity spread values are similar through both methods. However, for cases in which there is a slow-moving continuous mass, such as in cytoplasm, DPS tends to yield lower values for the diffusion coefficient.^31^

DPS has been applied successfully for label-free characterization of intracellular mass transport phenomena in live cells.^78^ As stated previously, QPI enables larger durations of sample observation because of the lack of phototoxicity. This property of QPI enables the monitoring of the dynamics over large durations using DPS. An example is reported in Ref. 79 in which microtubule dynamics were measured for 2 h.

## 4 Applications

In this section, we discuss some additional applications of QPI techniques related to monitoring and characterizing the dynamics of biological samples.

### 4.1 Membrane Dynamics

**Fig. 20:**
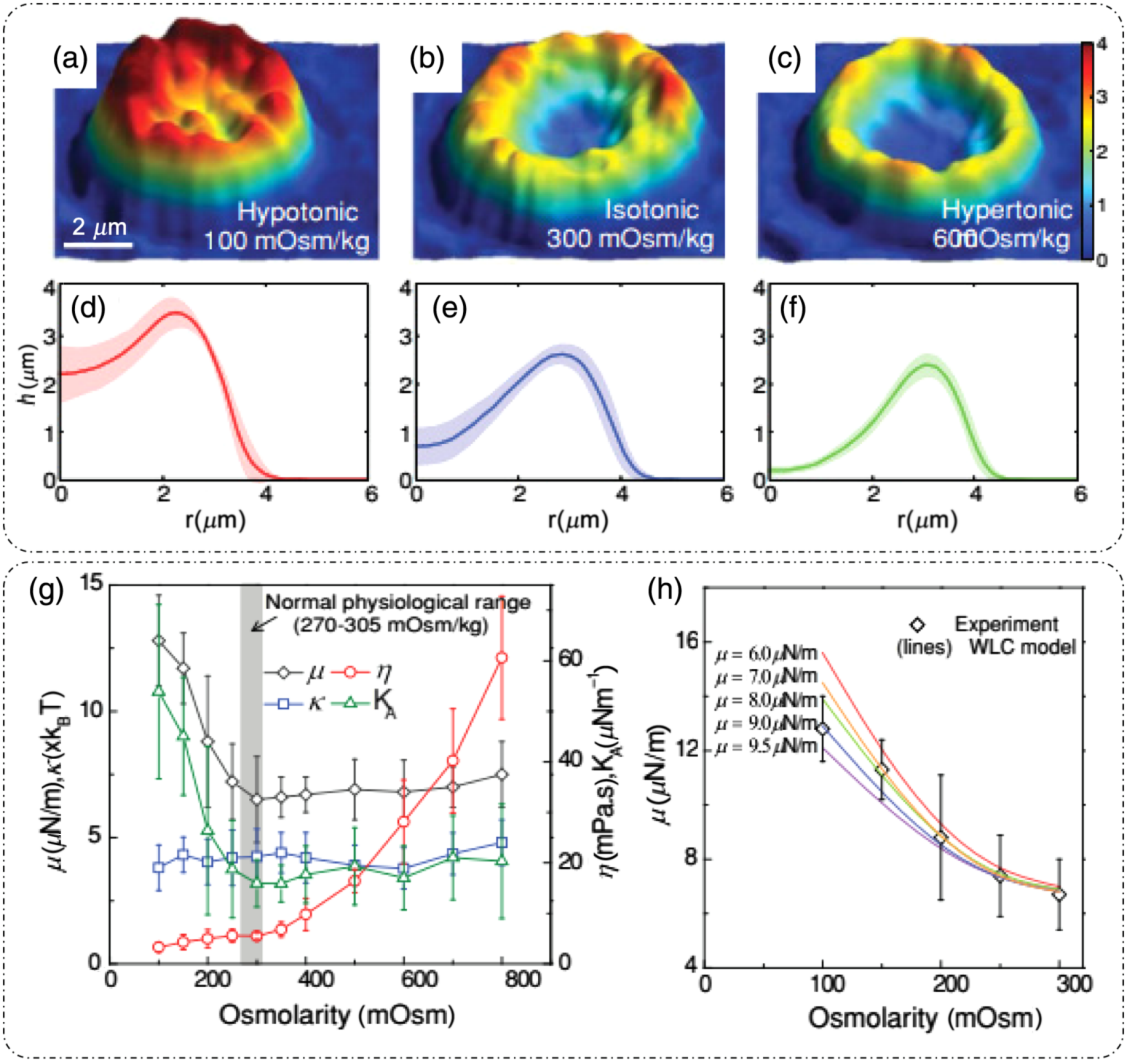
Applications: cellular dynamics measurement through DPM: (a)–(c) Thickness profiles of RBCs placed in solutions of varying osmolality. (d)–(f) Corresponding membrane height profiles versus distance from the center of the cells. (g) Extracted mechanical properties (shear modulus, cytosol viscosity, bending modulus, area compression modulus) of RBC membranes at different osmolarity levels. (h) Comparison of experimentally determined shear modulus μ with WLC with varying osmolarity. (a)–(h) Reproduced with permission from Ref. 80, © 2011, American Physical Society.

QPI has been applied extensively to study membrane dynamics. Of particular interest is the membrane of RBCs, which is composed of a network of spectrin and lipid bilayer.^80^ In Ref. 80, RBCs were placed in solutions of different osmolality levels to study the elasticity of the membrane. Figures 20(a)–20(c) represent the thickness profile calculated from the phase measurements using DPM for RBCs kept in three different solutions.^80^ When placed in a hypotonic solution, the cell swells up due to the influx of water [Fig. 20(a)].^80^ In an isotonic solution, no change is expected [Fig. 20(b)], and in a hypertonic solution, the cell shrinks due to the efflux of water from the cell [Fig. 20(c)].^80^ The membrane height fluctuations corresponding to Figs. 20(a)–20(c) are shown in Figs. 20(d)–20(f).^80^ Park et al.^80^ also noticed that the root mean square (RMS) membrane fluctuations, calculated by subtracting the time-average height profile image from each frame, are largest when the cell is placed in a normal osmolality solution and the fluctuations are diminished in the case of each hypo and hypertonic solution, displaying a reduced deformability of RBCs under stress.^80^ Based on the spatial correlations of membrane fluctuations and a continuum model of the spectrin-bilipid layer membrane, the mechanical parameters of the RBC membrane that include shear modulus, bending modulus, and area compression modulus as well as the viscosity of the cytosol were extracted in Ref. 80, as shown in Figs. 20(g) and 20(h) under varying osmolality conditions.^80^

**Fig. 21:**
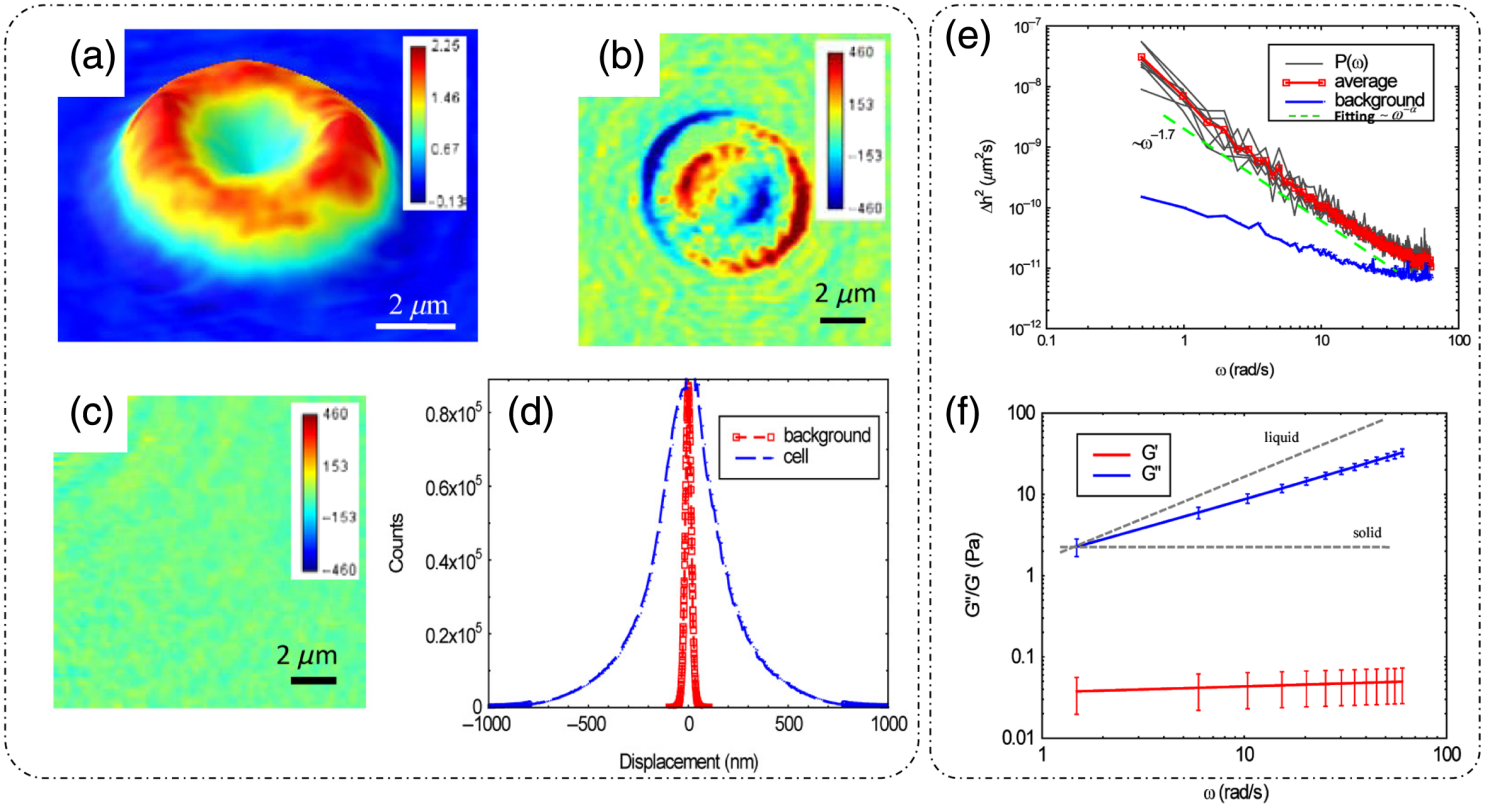
Applications: 3D dynamics by DPM: (a) average thickness distribution of an RBC with the color bar in micrometers. (b) RBC displacement map at one time-point with the color bar in nanometers. (c) Background displacement map with the color bar in nanometers. (d) Histograms of cellular and background displacements. (e) Membrane fluctuations power spectrum evaluated for seven RBCs. (f) Extracted viscous and elastic modulus versus frequency curves for RBCs. (a)–(f) Reproduced with permission from Ref. 81, © 2011, Optica.

An example of 3D RBC membrane dynamics measurement via DPM is shown in Fig. 21.^81^ Figure 21(a) shows the average thickness distribution of RBC with the corresponding displacement map at a particular time instant shown in Fig. 21(b).^81^ As discussed earlier, DPM has a high spatial and temporal sensitivity. The background displacement map shown in Figs. 21(c) and 21(d) is a comparison of histograms of cell and background displacements being well differentiated owing to the high optical path length sensitivity of DPM.^81^ The viscoelastic modulus was extracted using the fluctuation-dissipation theorem to connect membrane fluctuations to the viscous response in Ref. 81. The power spectra for seven RBCs are shown in Fig. 21(e), and the viscous and elastic modulus dependence on frequency is shown in Fig. 21(f).^81^ These trends imply the viscoelastic nature of RBCs.^81^

**Fig. 22:**
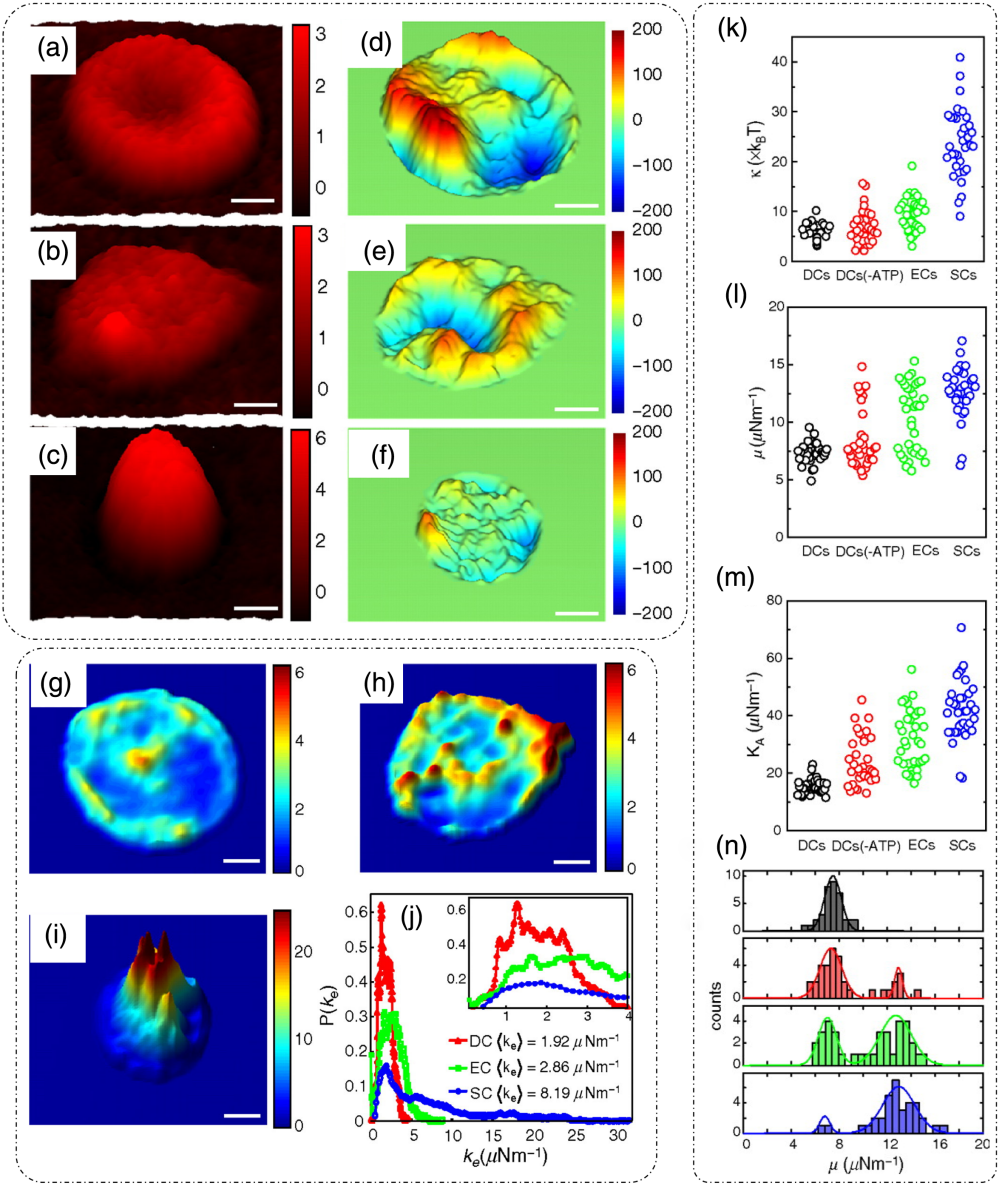
Erythrocyte dynamics through DPM: (a)–(c) thickness profile, the color bar in micrometers. (d)–(f) Corresponding instant displacement maps, the color bar in nanometers. (g)–(i) Effective local spring constant maps with the color bar in $\mu {\mathrm{Nm}}^{-1}$ for different types of RBCs-normal discoid, EC, and abnormal spherocytes, respectively. Scale bars for panels (a)–(i) indicate 1.5  μm. (j) Histograms of panels (g)–(i) with inset showing zoomed-in curves near origin. (k) Bending modulus. (l) Shear modulus. (m) Area modulus for different classes of RBCs. (n) Histograms show a bimodal distribution of shear modulus for all subtypes of RBCs in the study except normal discoid. (a)–(n) Reproduced with permission from Ref. 82, © 2010, PNAS.

Membrane fluctuations and changes in mechanical properties of RBCs, monitored and quantified using DPM as they transition from healthy morphology to unhealthy morphology, are discussed in Ref. 82. Figures 22(a–c)–22(d–f) show the thickness profile and instant displacement maps, respectively, for normal discoid, abnormal echinocyte (EC), and abnormal spherocyte RBC measured through DPM.^82^ In Ref. 82, under thermal equilibrium, each point on the membrane was expressed as an independent harmonic oscillator, having an effective local spring constant expressed in terms of the mean squared height fluctuations as $k_{e}=k_{B}T/\langle \Delta h^{2}\rangle$. The $k_{e}$ map for all three categories of RBCs is shown in Figs. 22(g)–22(i),^82^ and the histogram is shown in Fig. 22(j), where it can be seen that the mean $k_{e}$ is highest for spherocytes, implying increasing stiffness in the unhealthy RBCs.^82^ Based on a mathematical formulation described in Ref. 82, Park et al. extracted the bending, shear, and area modulus from curve fitting the theoretical model to the measured correlations of height fluctuations between points on the membrane separated in time and space.

Figures 22(k)–22(m)^82^ show the bending, shear, and area modulus for normal discoid (DC), discoid with ATP depletion (DC-ATP), abnormal echinocyte EC, and abnormal spherocyte (SC) RBC, respectively. All three moduli show an increase in degrading cell structure. The distribution of shear moduli shows that, with the exception of DC, the cell types have bimodal distributions [Fig. 22(n)] with one peak at a higher mean indicating increasing stiffness.^82^ Park et al.^82^ inferred that each abnormal cell has two types of spectrin networks, soft and stiff, as indicated by the bimodal distribution.^82^

**Fig. 23:**
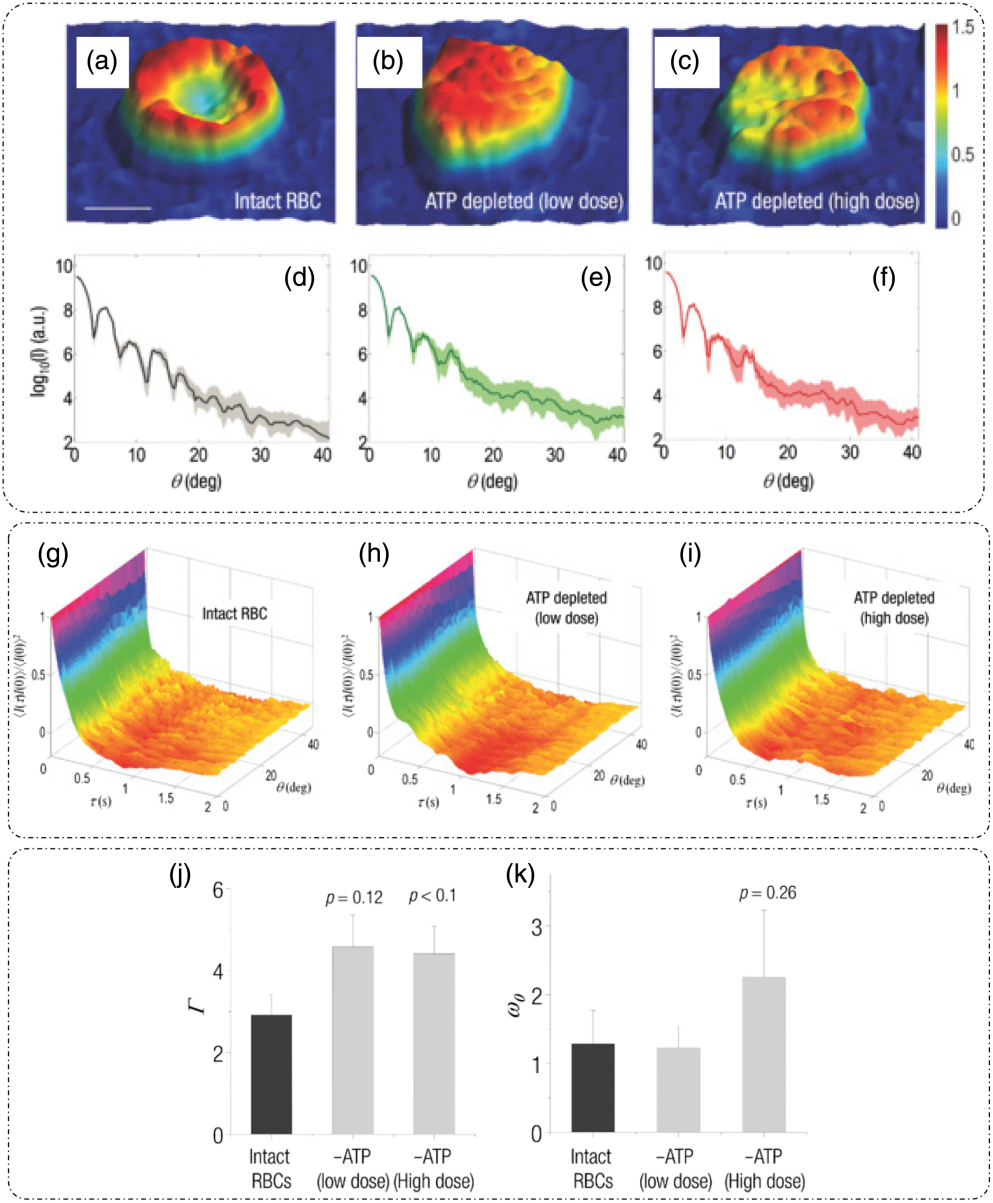
FTLS of erythrocyte: (a)–(c) time-averaged thickness profiles of RBCs under normal and ATP depletion conditions; the color bar is in micrometers, and the scale bars for panels (a)–(c) indicate 5  μm. (d)–(f) Corresponding angular scattering intensity curves and (g)–(i) normalized temporal intensity autocorrelation function versus scattering angle and decay rate plot for healthy RBCs, ATP-depleted RBCs with a low dose of inosine and iodoacetamide, and ATP-depleted RBCs with a high dose of inosine and iodoacetamide. (j), (k) The line width or the bandwidth is statistically different between ATP depleted (high dose) and normal RBC, while there is no such difference in peak frequency. (a)–(k) Reproduced with permission from Ref. 83, © 2011, SPIE.

FTLS has also been used to study the light scattering process of RBCs.^83^ Figures 23(a)–23(c) show the time averaged thickness profile of an RBC undergoing ATP depletion, resulting in a loss of central concave cavity.^83^ The corresponding angular scattered intensity plots are shown in Figs. 23(d)–23(f), showing a loss of oscillatory behavior at higher scattering angles as the cell undergoes ATP depletion.^83^ For DLS measurements, fast time-lapse imaging was performed for 2 s collecting 240 frames.^83^ The temporal autocorrelation function of scattered intensity was then calculated from the scattered fields obtained using the FTLS procedure.^83^ Plots of the normalized temporal intensity autocorrelation function versus scattering angle and decay rate are shown in Figs. 23(g)–23(i).^83^ Assuming a near-Lorentzian power spectrum, the autocorrelation follows a damped cosine function,^83^ and the peak frequency $\omega_{0}$ and bandwidth Γ were extracted by a curve fit.^83^ It was shown in Ref. 83 that the bandwidth Γ varies significantly between healthy and ATP-depleted RBCs, and the change in mean frequency $\omega_{0}$ was not statistically significant, as shown in Figs. 23(j) and 23(k). It was also reported that the ATP-depletion causes reversible changes in RBC mechanics.^83^ Changes in the tension coefficient during the structural transition from discocytic to spherocytic shape have been quantified using stabilized HPM (discussed earlier in part I of this review^8^) in Ref. 84. The spatial and temporal coherence of RBC membrane motions has been studied and characterized using DPM, and viscoelastic moduli were extracted based on DPM data in Ref. 85. A review of RBC dynamics studied through QPI can be found in Ref. 86.

**Fig. 24:**
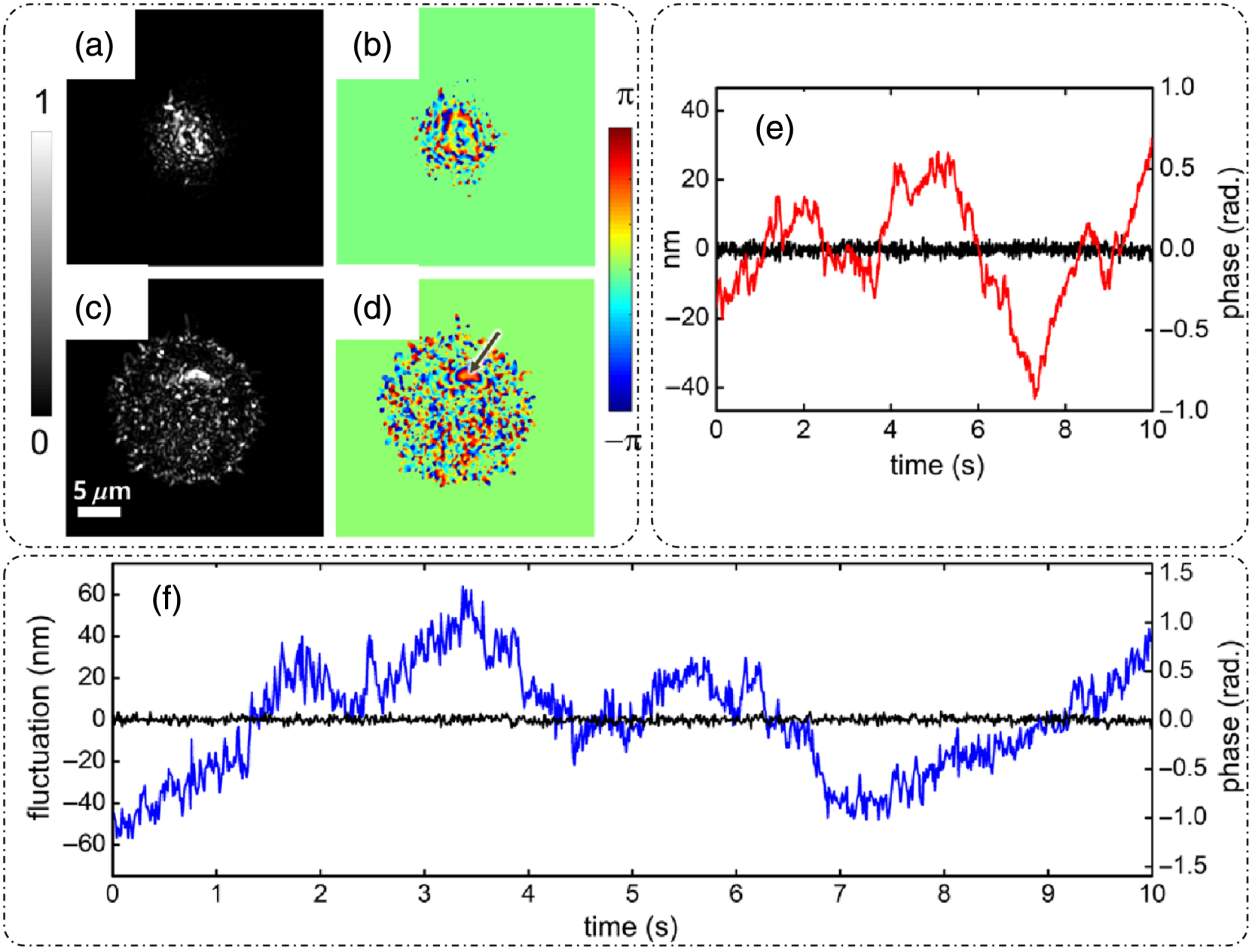
RPM: measured reflection intensity and phase map for (a), (b) plasma membrane and (c), (d) nuclear membrane, (e) temporal background noise before (red) and after (black) noise stabilization. (f) Temporal fluctuations of the nuclear membrane at the point indicated by an arrow in panel (d) with the black line indicating the background. The color bar for the intensity maps indicates normalized intensity, and that for the phase maps indicates radians. (a)–(f) Reproduced with permission from Ref. 88, © 2018, Optica.

Reflection phase microscopy (RPM) is another QPI configuration that combines low-coherence interferometry with full-field off-axis DHM.^87^ This method provides a single-shot, full-field quantitative phase information.^87^ Choi et al.^88^ introduced another variant of RPM that has an improved depth sectioning capability and hence a better axial resolution (640 nm).^88^ The sensitivity of optical path length detection was reported to be down to 1 nm.^88^ This was possible due to the utilization of spatio-temporal coherence gating induced using a broadband supercontinuum laser source and a rotating diffuser for dynamic speckle illumination.^88^ This technique enabled high-speed (100 fps, due to the single-shot nature of measurement) and highly sensitive characterizations of nuclear membrane dynamics of eukaryotic cells.^88^ Figure 24 shows the dynamic characterization of the nuclear membrane of human cancer cells, with reflection intensity and phase images for plasma membrane [Figs. 24(a) and 24(b)] and nuclear membrane [Figs. 24(c) and 24(d)].^88^ Using a phase stabilization technique that involves the estimation of phase noise from a reference region by slightly tilting the sample plane, the background noise was reduced from the red curve to the black line [Fig. 24(e)].^88^ Finally, the nuclear membrane fluctuations for the spatial location indicated by an arrow in Fig. 24(d), acquired at the speed of 100 fps, are shown in Fig. 24(f).^88^

**Fig. 25:**
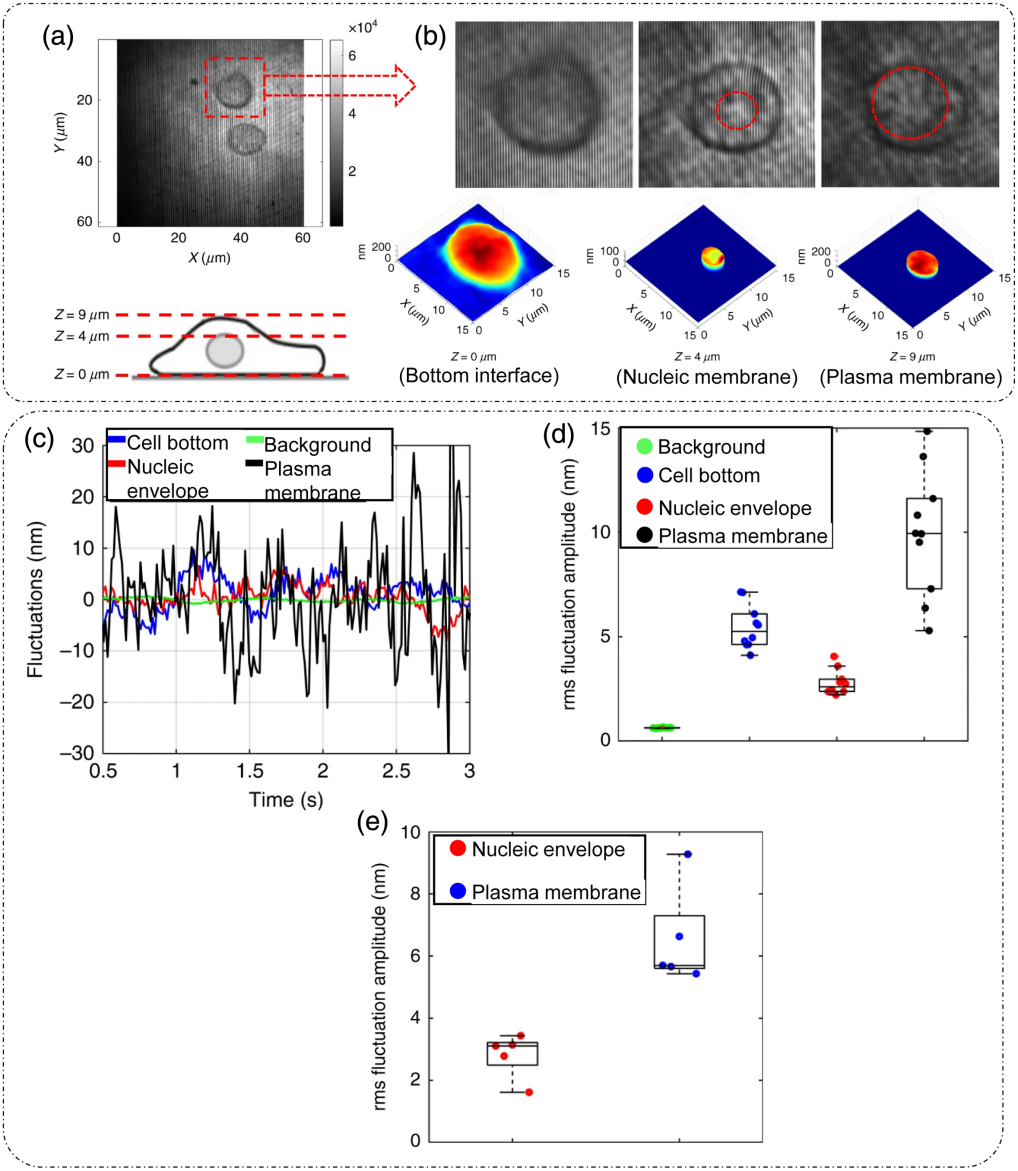
Confocal reflectance interferometric microscopy of mouse embryonic stem cells: (a) interferometric detection at a cell-dish interface (z=0  μm) with three different depths (cell-dish interface, nucleic envelope, and plasma membrane) indicated in the diagrammatic view of a cell below. (b) Zoomed-in interferograms of the three depths (top row) and the corresponding 3D height map (bottom row). (c) Temporal membrane fluctuations amplitude for background and the three depths. (d) Boxplots showing the RMS fluctuations amplitudes for the background and the three depths for one cell. (e) RMS membrane fluctuation amplitudes of the nucleic envelope and plasma membrane for five cells. (a)–(e) Reproduced from Ref. 89, under CC BY license.

A near common-path, DPM-based, confocal reflectance interferometric microscopy was introduced by Singh et al.^89^ with a reported optical path length sensitivity of 0.2 nm. This technique involves an interesting modification in which a scan grid of confocal spots is generated using a DMD, thereby enabling a full-field acquisition with confocal-like quality.^89^ Figure 25 shows the dynamic characterization of different parts of a mouse embryonic stem cell (depicted in Fig. 25(a) showing the full interferogram) measured through this technique.^89^ Interferograms and the corresponding 3D height maps for the three parts (cell bottom, nucleic envelope, and plasma membrane) are shown in Fig. 25(b).^89^ The temporal fluctuations [Fig. 25(c)] and the RMS fluctuation amplitude [Fig. 25(d)] for the three parts of the same cell reveal the differences in their dynamics, with the nucleic envelope having a lower RMS fluctuation amplitude than the plasma membrane, which implies that the nucleic envelope has a higher stiffness.^89^ The authors of Ref. 89 also repeated the observations on multiple cells and observed a similar trend in the membrane dynamics [Fig. 25(e)].^89^

### 4.2 Intracellular Transport

Intracellular mass transport in a beating cardiomyocyte cell imaged using SLIM was measured through DPS in Ref. 90. That study reported that the transport was deterministic in nature, and the speed distribution yielded almost identical widths in different cells implying, that the cells were synchronized in motion.^90^ Another study conducted on mass transport in neurites using SLIM and DPS found that the bidirectional movement of cargo inside the neurites can be characterized by a one-dimensional diffusion–advection equation and that such a transport is essentially deterministic with an approximately zero mean velocity.^91^ DPS has also been applied to determine 3D mass transport in the neurons in both horizontal and vertical directions.^92^ It was also shown that the cell bodies exhibit higher spread in the advection velocity distribution compared with neurites, with the spread being more pronounced and significant in the YZ (vertical) plane than the XY (horizontal) plane.^92^

### 4.3 Cytoskeleton

**Fig. 26:**
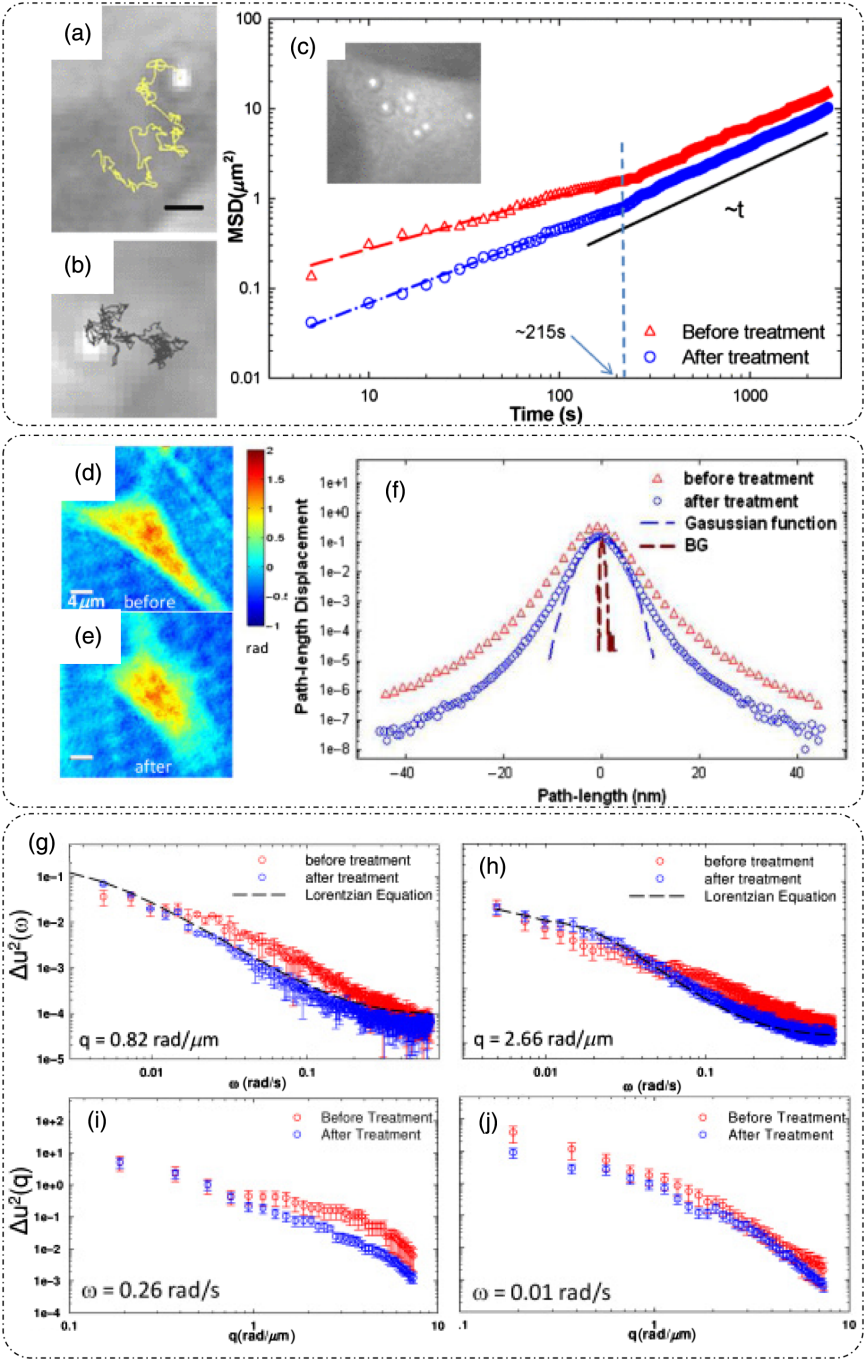
Cytoskeleton dynamics through FTLS: (a), (b) Trajectories of beads attached to the glial cell before and after Cyto-d treatment, respectively. (c) Temporal MSD curves of the beads. (d), (e) Phase maps of a glial cell before and after treatment with Cyto-d, respectively. (f) respective path length displacement histograms; BG, background. (g), (h) Temporal power spectra for two distinct spatial frequencies. (i), (j) Spatial power spectra at two different temporal frequencies. (a)–(j) Reproduced with permission from Ref. 93, © 2010, Optica.

Actin plays a diverse role in cellular motility and membrane dynamics. Dynamics of the cytoskeleton at the single-cell level were measured and quantified using FTLS in Ref. 93. They studied active dynamics in enteric glial cells driven by actin. FTLS measurements involved the acquisition of 512 frames at a rate of 0.2 fps before and after treating the cells with cytochalasin d. Cyto-d is a chemical that inhibits the actin polymerization/depolymerization process and in turn induces fragmentation of the actin filament.^93^ A control experiment based on particle tracking was also conducted; polystyrene beads were attached to the glial cell membrane and the MSD averaged over time, and particles were measured before and after treatment with Cyto-d.^93^ Figures 26(a) and 26(b) show the displacement trajectories for beads attached to the membrane before and after the Cyto-d treatment, showing a marked reduction in displacement after inhibiting actin.^93^ The time trend of MSD before and after the drug treatment is shown in Fig. 26(c), where after a cut-off time point of ∼200  s, the curve changes slope for both trends.^93^ The exponents before this cut-off for both treated and untreated cells exhibit sub-diffusion motion characteristics, but after the cut-off, the actin-inhibited trajectory follows a curve with a linear dependency on time indicative of a Brownian motion.^93^ The phase profile of a single glia cell before and after treatment with Cyto-d is shown in Figs. 26(d) and 26(e), and the path length displacement histogram is shown in Fig. 26(f).^93^ The changes in the optical path length observed in the histograms in Fig. 26(f) can be attributed to local thickness changes due to cell membrane dynamics or local RI changes indicative of mass transport within the cell.^93^ It can be seen that the displacements are reduced after treatment as confirmed by particle tracking results in Fig. 26(c), indicating that actin is a major factor in cell motility.^93^

The effect of actin inhibition captured by FTLS is shown in Figs. 26(g)–26(j).^93^ Figures 26(g) and 26(h) show the spatially averaged power spectra at two different wavenumbers for both before and after treatment cases. After treatment, the power spectra at both q remain essentially similar, exhibiting a Brownian motion characterized by the Lorentzian distribution fit. However, that is not the case with cells before treatment where actin is active. The temporal effects on scattering are demonstrated through the temporally averaged power spectrum at two different temporal frequencies, as illustrated in Figs. 26(i) and 26(j).^93^ For lower frequencies [Fig. 26(j)], the power spectra are similar. However, for higher frequencies [Fig. 26(i)], they differ considerably. It was concluded that this may be indicative of the actin polymerization lifetime.^93^

Thus, enabled by techniques such as FTLS, DPS, and DLS, the dynamics of live cells can be monitored in a non-invasive and label-free manner. This could be very useful for estimating the *in vitro* cellular behavior.

## 5 Current Limitations and Future Directions

Although QPI techniques provide a great advancement in optical imaging, there are some aspects that require more development. For example, there remains a need for improved QPI techniques that can circumvent the diffraction limit of resolution and increase the amount of information that can be extracted from highly turbid 3D samples through the enhanced suppression of the higher-order scattering.^94^ Another major limitation of 3D QPI is the missing cone problem, which causes reconstruction artifacts along the axial direction and needs more algorithmic developments. Advanced machine learning methods are expected to deliver improved performance in solving the missing cone problem.^95^^,^^96^ Yet another limitation of QPI techniques is the lack of chemical specificity. Chemical specificity is of utmost importance in biology for recognizing chemical constituents or proteins. Although intrinsic measures such as dry mass and dry mass density convey information about the overall protein content, individual protein identification via their chemical signatures is currently not feasible using QPI alone. Hence, a computational multimodal machine learning system incorporating QPI and data from other chemically specific spectroscopic techniques can extend the utilization of such imaging modalities to the basic science community. Super-resolution fluorescence imaging can also benefit from QPI techniques in which 2D/3D context information is required.^97^ Most of the 3D QPI techniques are based on weak scattering approximations, which may not be best suited for highly scattering samples such as organoids or spheroids. A greater understanding of inverse-scattering problems under multiple scattering conditions and algorithmic developments incorporating multiple scattering phenomena represents another promising area for future research.^98^^–^^101^ Increasing the throughput and hence improving the temporal resolution of measurements using a combination of sparse measurements and machine learning approaches^58^ also require more investigations. These developments will eventually help QPI to be translated to and become a powerful tool in clinical settings.

## 6 Conclusion

QPI techniques have seen tremendous growth in the past few decades owing to their label-free, non-invasive, and quantitative phase/RI measurement capability.^1^^,^^14^ The intrinsic measures based on phase measurements have introduced a range of new information to researchers as potential biomarkers.^19^^,^^20^^,^^102^^–^^107^ In these reviews, we aimed to cover the basic principles and technical aspects of instrumentation along with a discussion of applications for both 2D and 3D QPI techniques. Several realizations of such 2D/3D QPI techniques were discussed. Methods to characterize cellular and sub-cellular dynamics were also reviewed.

## Data Availability

No new data were generated in this study. No codes were used for this study.
